# Entity and relation extraction from clinical case reports of COVID-19: a natural language processing approach

**DOI:** 10.1186/s12911-023-02117-3

**Published:** 2023-01-26

**Authors:** Shaina Raza, Brian Schwartz

**Affiliations:** 1 Public Health Ontario (PHO), Toronto, ON Canada; 2 Dalla Lana School of Public Health, University of Toronto, Toronto, ON Canada

**Keywords:** Natural language processing, Data cohort, COVID-19, Named entity, Relation extraction, Transfer learning, Artificial intelligence

## Abstract

**Background:**

Extracting relevant information about infectious diseases is an essential task. However, a significant obstacle in supporting public health research is the lack of methods for effectively mining large amounts of health data.

**Objective:**

This study aims to use natural language processing (NLP) to extract the key information (clinical factors, social determinants of health) from published cases in the literature.

**Methods:**

The proposed framework integrates a data layer for preparing a data cohort from clinical case reports; an NLP layer to find the clinical and demographic-named entities and relations in the texts; and an evaluation layer for benchmarking performance and analysis. The focus of this study is to extract valuable information from COVID-19 case reports.

**Results:**

The named entity recognition implementation in the NLP layer achieves a performance gain of about 1–3% compared to benchmark methods. Furthermore, even without extensive data labeling, the relation extraction method outperforms benchmark methods in terms of accuracy (by 1–8% better). A thorough examination reveals the disease’s presence and symptoms prevalence in patients.

**Conclusions:**

A similar approach can be generalized to other infectious diseases. It is worthwhile to use prior knowledge acquired through transfer learning when researching other infectious diseases.

**Supplementary Information:**

The online version contains supplementary material available at 10.1186/s12911-023-02117-3.

## Introduction

The COVID-19 pandemic has generated massive amounts of clinical, behavioral, social, and epidemiological data. As of August 2, 2022, COVID-19 has infected more than 578 million people, with over 6.4 million deaths [1]. There are serious concerns regarding the impact of the infectious disease on society, global health, and the economy [2–4]. It is necessary to develop an efficient surveillance system that can track the spread of infectious diseases by collecting, analyzing, and reporting data to those responsible for preventing and controlling the disease.

Despite significant advances in informatics, some hurdles to studying infectious diseases remain unresolved. First, the conventional methods are typically trained on limited data [5]. In real-world scenarios, a substantial amount of clinical data is available as free-text [6] data, such as electronic health records (EHRs), or published literature [7]. Second, the existing methods mainly rely on manually labeled structured datasets for predictive modeling. Although governments and organizations can always collect data on real-world pandemic events, it is highly costly and time-consuming. Also, when these datasets are made available to the research community, reporting lags behind the current number of COVID-19 cases.

Given the aforementioned challenges, we propose a NLP framework that uses deep neural networks and transfer learning to automatically extract valuable information from texts for analyzing COVID-19 and other infectious diseases. Transfer learning [8] is a technique that allows models trained on one task to be applied to another related task, making it a powerful tool for NLP tasks. The main objective of this research is to bridge the gap that exists between NLP techniques and their applications in public health (PH). The specific contributions of this work are:

1. A data cohort is created by curating published COVID-19 case reports from the National Institutes of Health (NIH) source between March 1, 2022, and June 30, 2022. The data is parsed to create a COVID-19 database. A portion of the data is prepared for gold annotations, and the technique of active learning [9] is applied for corpus re-annotation.
2. A deep learning-based named entity recognition (NER) algorithm is proposed to learn the clinical (disease, condition, symptom, drug) and non-clinical (social determinants of health) concepts from the case reports data. Additionally, a relation extraction (RE) model for predicting relationships (such as disease-brings-complications, treatment-improve-condition, and drug-and-adverse effect) between entities is proposed. The performance of these approaches is evaluated through an extensive comparison with several baseline methods, including ML, deep learning, and Transformer-based models on various datasets. The results are discussed in the context of public health surveillance and monitoring. An empirical analysis of the proposed approach in extracting key information from the texts, along with a discussion of the benefits and limitations of this approach is also presented.

The current study improves upon previous efforts by extracting clinical and non-clinical entities from the case report data. The key contribution of this work is the integration of various NLP components into a pipeline structure, which enables the efficient extraction of valuable information from texts. The research question that guides this study is “How effective is transfer learning in enhancing the performance of NLP tasks for identifying and extracting information about infectious diseases in the clinical and public health domain?” Through this study, we aim to provide solutions that can assist policymakers in their decision-making processes and accelerate research on the subject.

## Related work

Named entity recognition (NER) [10] is a subtask of NLP that involves identifying and classifying named entities in text into predefined categories such as person names, organizations, locations, medical codes, etc. Biomedical NER [11] is a specialized NER task that focuses on identifying and classifying biomedical entities, such as genes, proteins, and diseases, in unstructured text. State-of-the-art biomedical NER models include BiLSTM-based [12] and Transformer-based [13–15] models, which can capture contextual dependencies and are robust to noise and variations in the input data. They can also be combined with other techniques such as attention mechanisms [16] and convolutional neural networks (CNN) [12] to further improve their performance. Recent developments in biomedical NER include the use of transfer learning [17], BERT-like [13], attention-based [18], multi-task learning [19], and hybrid models [20] to improve the performance of these models.

Relation extraction (RE) [21, 22] is the process of identifying and classifying relationships between entities in text. Statistical and machine learning (ML) methods [23], such as rule-based systems, SVMs, and decision trees, can be used for RE tasks, although they may struggle with more complex relationships. Deep learning models, such as CNN [24] and recurrent neural networks (RNN) [25], can be used for RE tasks and can handle complex relationships. These deep neural network-based models usually require a large amount of labeled training data and computational resources.

Zero-shot learning (ZSL) [26] is an ML problem in which a learner observes samples from classes that were not observed during training and must predict which class they belong to. The topic of ZSL and RE in combination has received relatively little research to date. One such study [27] manually creates templates of new-coming unseen relations, while the other study [28] treats the zero-shot prediction as the text entailment task. Some other works [29] consider ZSL by leveraging knowledge gained from BERT-like models [30] to predict unseen relations without manual labeling, which is also a motivation in this research.

### Comparison with related works

Several works focus on extracting clinical information, particularly related to COVID-19, from unstructured text data. For example, Lybarger et al. [31] present a corpus of EHRs from COVID-19 patients with annotations for diagnoses, symptoms, and other clinical events. They propose a neural event extraction framework using a BiLSTM-CRF model for identifying and classifying these events. Luo et al. [32] propose a Transformer architecture trained on a large annotated dataset of COVID-19 symptoms. CORD-19 [33] is another large dataset of COVID-19 research papers compiled by Kaggle that can be used for tasks such as information extraction and text classification. Silverman et al. [34] present a NER model based on a BiLSTM-CNN architecture for extracting symptoms from unstructured COVID-19 data. These works have the potential to be used for tasks such as public health surveillance and monitoring. In recent works, relations from texts are typically extracted using statistical methods such as decision trees [35]. Recently, deep neural networks such as BiLSTM-based models CRF [12, 36, 37] and BERT-like methods [13, 38] have also been used to extract relations, which are both very robust and accurate but require a large amount of labeled data.

In contrast to earlier studies [39–41], our NER method also identifies non–clinical factors like social determinants of health (SDOH) [38] in addition to a variety of clinical factors. This is significant because SDOH factors have a significant impact on health outcomes, particularly during a pandemic like COVID-19. Additionally, our RE method extracts relationships from clinical texts without the need for labeled data, which sets it apart from existing works [13, 42] that require labeled data. We have combined ZSL, transfer learning, and RE in the context of COVID-19 to offer a comprehensive approach to understand the impact of the pandemic on population health. We have thoroughly tested and optimized our method through ablation studies to ensure maximum effectiveness.

## Materials and methods

In this study, we propose an NLP architecture for extracting key information from case reports data. This architecture has three layers: a data layer, which is responsible for preprocessing, preparing, and annotating the text data; an NLP layer, which includes a NER module to extract named entities (e.g. diseases, symptoms, conditions, social determinants of health) from the data and a RE module to infer relationships between the entities (such as disease-symptoms relationships, etc.); and an evaluation layer, which is used to evaluate the performance of the NLP modules and to assess the effectiveness of the proposed methods through empirical analysis.

### Data cohort preparation

In this study, we create a data cohort from electronic case reports of COVID-19 patients. A case report [43] is a published article describing a patient’s disease, diagnosis, treatment, symptoms, or therapy. We curated the case reports using a search query (Additional file 1: Table S1) using the NIH National Library of Medicine (NLM) [44] API that allowed us to get case reports from various journals. These case reports comply with CARE (CAse REports) principles [45], which specify that case reports should not contain any patient-identifiable information.

#### Inclusion and exclusion criterion

We consider the case reports to meet the eligibility criteria in Table 1. The exclusion criteria for this study are as:

- Grey literature, preprints, and clinical trials are excluded.
- Non-English content is excluded. Evidence suggests that excluding non-English publications from evidence synthesis does not bias conclusions [46].

**Table 1:** Selection criteria of the data

Criteria	Description
Population	Child: 6–12 years, Adolescent: 13–18 years, Adult: 19–44 years, Middle Aged: 45–64 years, Aged: 65 + years
Concept	Any approaches related to clinical classification or interventions, including screening, diagnosis, treatments, and therapies for COVID-19 with a focus on long-COVID conditions
Study design	Case reports
Timeframe	1st March 2021–30th June 2022
Language	English

After applying these filtrations, we obtained 4338 case reports

### Proposed framework

**Fig. 1:**
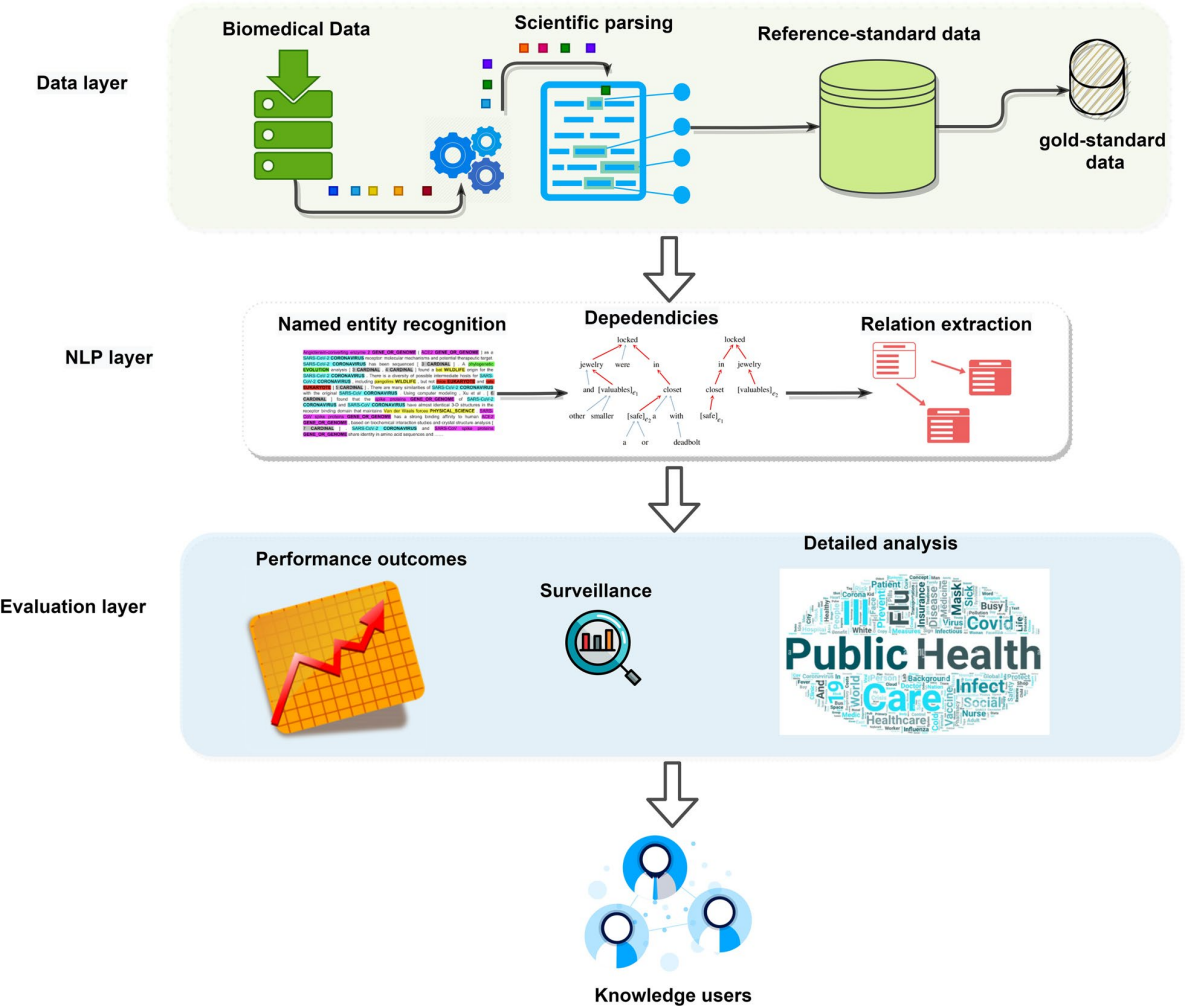
Proposed framework for pandemic surveillance

The proposed framework is shown in Fig. 1 and explained next.

#### Data layer

We began by gathering the biomedical data, which are COVID-19-related case reports from NIH sources (data cohort discussed above). The biomedical data goes into the scientific parser. We used the Spark OCR [47] to parse the case reports’ PDFs and extracted the content in a data frame format, with each row corresponding to a case report (document) and a column containing extracted text from the PDF texts. The parsed documents were indexed using Elasticsearch [48], which helps with speedy document retrieval, to create a reference-standard dataset. A *reference-standard dataset* [49], generally, refers to the collection and compilation of primary and secondary data sources that can be re-used and cited for various purposes, such as biomedical data retrieval.

*Gold-standard data* A random portion of the data (200 case reports) was issued to create a gold-standard dataset (manually annotated corpus) [50]. We draw inspiration from a previous work [51] on case reports annotations and consider only the case report texts rather than complete manuscript texts for this data preparation. Four experts from a biomedical domain annotated around 200 case reports with the clinical and non-clinical named entities. We use the Spark NLP annotation tool [52] to annotate the chosen case reports with the named entities. These named entities are given in Additional file 1: Table S2.

We have drawn inspiration from the guidelines for annotating named entities in literature sources [53, 54]. For Inter-annotator agreement (IAA) [55], we employ the simple agreement method, which calculates the percentage of annotations that match among all annotators without considering random chance (as in Cohen and Fleiss’ kappa statistics). The guidelines for our annotation task are provided in Table S2. We save these annotations into the CONLL-2003 [56] format, a prototypical data format for NER tasks. At the end of this step, we found approximately 500 sentences and 3048 gold labels.

*Active learning* Since our models are based on deep neural networks, it is best to train with more labeled data. We used the *active learning* [57] technique (shown in Additional file 1: Figure S1) for more data labeling. We began this active learning loop with 500 sentences (from the gold-standard dataset) and added batches of 100 samples (reports) until it reached around 1000 samples, with the best accuracy of approximately 92.80%. By the end of this task, we obtained 47,888 sentences with approximately 387,899 named entities. We used this data to train our NER model.

*Task-specific Transformer model* We fine-tuned the Bidirectional Encoder Representations from Transformers (BERT) for Biomedical Text Mining (BioBERT) [13] using our annotated dataset to prepare a task-specific model, which is more lightweight than a typical task-agnostic model [58]. We release the weights of our fine-tuned model here [59] and show our task-specific transformer model in Additional file 1: Figure S2.

#### NLP layer

We develop two NLP models in this layer, which are (1) a NER module to produce named entities; (2) a RE module to define relationships between the named entities.

**Fig. 2:**
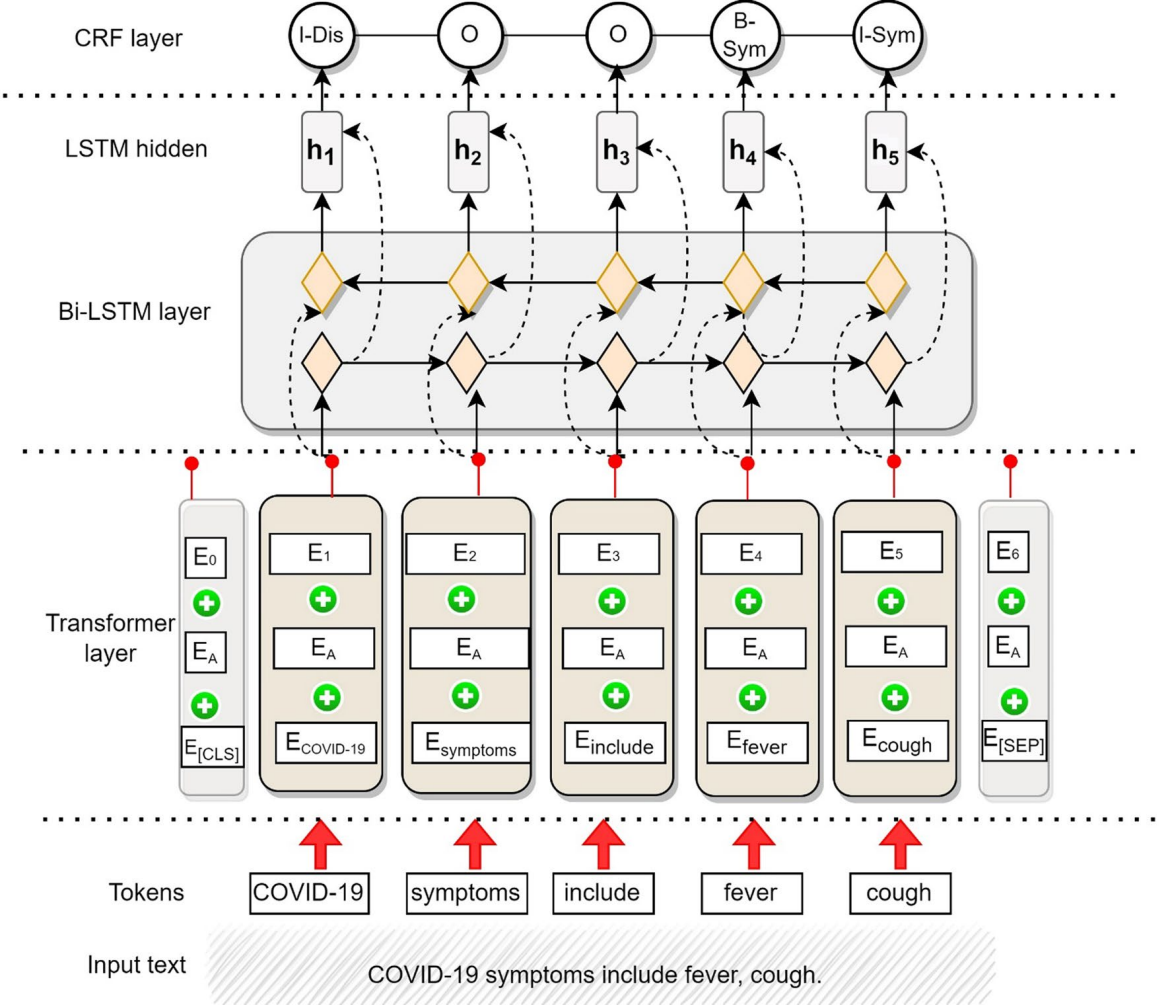
Proposed model for named entity recognition

*Named entity recognition model* This model is inspired by bi-directional (Bi) long short-term memory (LSTM) model with a conditional random field (CRF) layer [60], but we add a Transformer layer to produce a variant of the model. We show our Transformer-BiLSTM-CRF model in Fig. 2 and explain its working next. The notations in formulae are given in Additional file 1: Table S3.

*Transformer layer* The input text sequence (shown in the bottom layer of Fig. 2) goes into the Transformer layer. In this layer, we adapt the BERT architecture for the embedding. The core of BERT is implemented by multi-layer Transformer encoders [61] which is dependent on the self-attention mechanism. The self-attention information in this layer is obtained by formula (1) [61]:

1 $$\begin{array}{*{20}c} {attention\left( {Q,K,V} \right) = softmax\left( {\frac{{QK^{T} }}{{\sqrt {d_{k} } }}} \right)V} \\ \end{array}$$

The output of this layer is word embeddings in the form of word vectors, which are fed into the next layer, the BiLSTM layer, to learn context features. Our technical contribution here entails the use of our task-specific Transformer model for extracting these entities.

*BiLSTM layer* The Transformer output vector is input to the BiLSTM layer. The BiLSTM units receive a dynamic sequence of word vectors as input and learn to extract local features from the sentence. The forward LSTM model generates hidden state sequences, and the backward LSTM model combines them to produce the complete hidden state sequence for the sentence sequence. The related information is obtained by the formula (2) [62].

2a $$\begin{array}{*{20}c} {i_{t} = \sigma \left( {W_{i } x_{t} + W_{hi } h_{t - 1} + b_{i} } \right)} \\ \end{array}$$

2b $$\begin{array}{*{20}c} {f_{t} = \sigma \left( {W_{f } x_{t} + W_{hf } h_{t - 1} + b_{f} } \right)} \\ \end{array}$$

2c $$\begin{array}{*{20}c} {o_{t} = \sigma \left( {W_{o } x_{t} + W_{ho } h_{t - 1} + b_{o} } \right)} \\ \end{array}$$

2d $$\begin{array}{*{20}c} {\tilde{C}_{t} = tanh\left( {W_{C } x_{t} + W_{hC } h_{t - 1} + b_{C} } \right)} \\ \end{array}$$

2e $$\begin{array}{*{20}c} {C_{t} = f_{t} \otimes C_{t - 1} + i_{t} \otimes \tilde{C}_{t} } \\ \end{array}$$

2f $$\begin{array}{*{20}c} {h_{t } = o_{t} *\tanh \left( {C_{t - 1} } \right)} \\ \end{array}$$

The input *x* to the BiLSTM layer is the output of the Transformer. The BiLSTM layer uses both a forward and backward LSTM to capture contextual information and obtain global features for each moment in the input sequence. This allows the BiLSTM to effectively process the input sequence and extract useful features from it. The output of the BiLSTM layer is a sequence of hidden states, one for each input word.

*CRF layer* The input to the CRF layer is the output sequence of the BiLSTM layer. The CRF layer captures the dependency relationship between the named tags and constrains them to the final predicted labels [63]. The conditional probability distribution in CRF is represented by $P(Y|X)$ and shown in formula (3) [64].

3 $$\begin{array}{*{20}c} {p\left( {y{|}x} \right) \propto \exp \left( {\mathop \sum \limits_{k = 1}^{K} \omega_{k} f_{k} \left( {y,x} \right)} \right)} \\ \end{array}$$

The output of the CRF layer is the Inside–Outside–Before (IOB) format, a scheme for tagging tokens in NER chunking tasks [65]. We convert the IOB representation of our model to a user-friendly format by associating chunks (recognized named entities) with their respective labels, as shown in Additional file 1: Figure S3. We also filter out the NER chunks with no associated entity (tagged as ‘O’). The model’s output is the named entities given in Additional file 1: Table S2.

We note a case report on long-COVID, titled: “Case report: overlap between long covid and functional neurological disorders” [66], and show the named entities extracted by our model in Additional file 1: Table S4. We also show the visual representation of named entities from the snippet of the case report in Additional file 1: Figure S4. This approach can also detect information from a non-COVID-19 case report [67], as shown in an example in Additional file 1: Table S5.

*Relation Extraction* A relation can be defined as a triple (shown in formula 4) with indices in ${s}_{1}$ and ${s}_{2}$ that delimit a named entity mentioned in *x* (sequence of tokens).

4 $$\begin{array}{*{20}c} {r = \left( {x,s_{1} ,s_{2} } \right)} \\ \end{array}$$

The RE task can identify a specific relation between two co-occurring entities [22], such as symptom-disease, disease-disease, and drug-effects associations. Prior to the RE, there is a dependency parsing (DP) task (example shown in Additional file 1: Figure S5), which refers to examining the dependencies between the words of a sentence to analyze its grammatical structure [68]. For instance, to identify the subjects and objects of a verb and the terms that modify (describe) the subject. These dependencies go as input to the RE module.

Taking inspiration from recent NLP works on RE [27, 69], we employ zero-shot learning (ZSL) [26] to infer relations from the texts. ZSL is an ML technique in which a model observes samples from classes which has not been explicitly observed before during training. We already our NERT model as a base for the RE model. The RE model can predict relations between named entities without any additional training through ZSL.

**Fig. 3:**
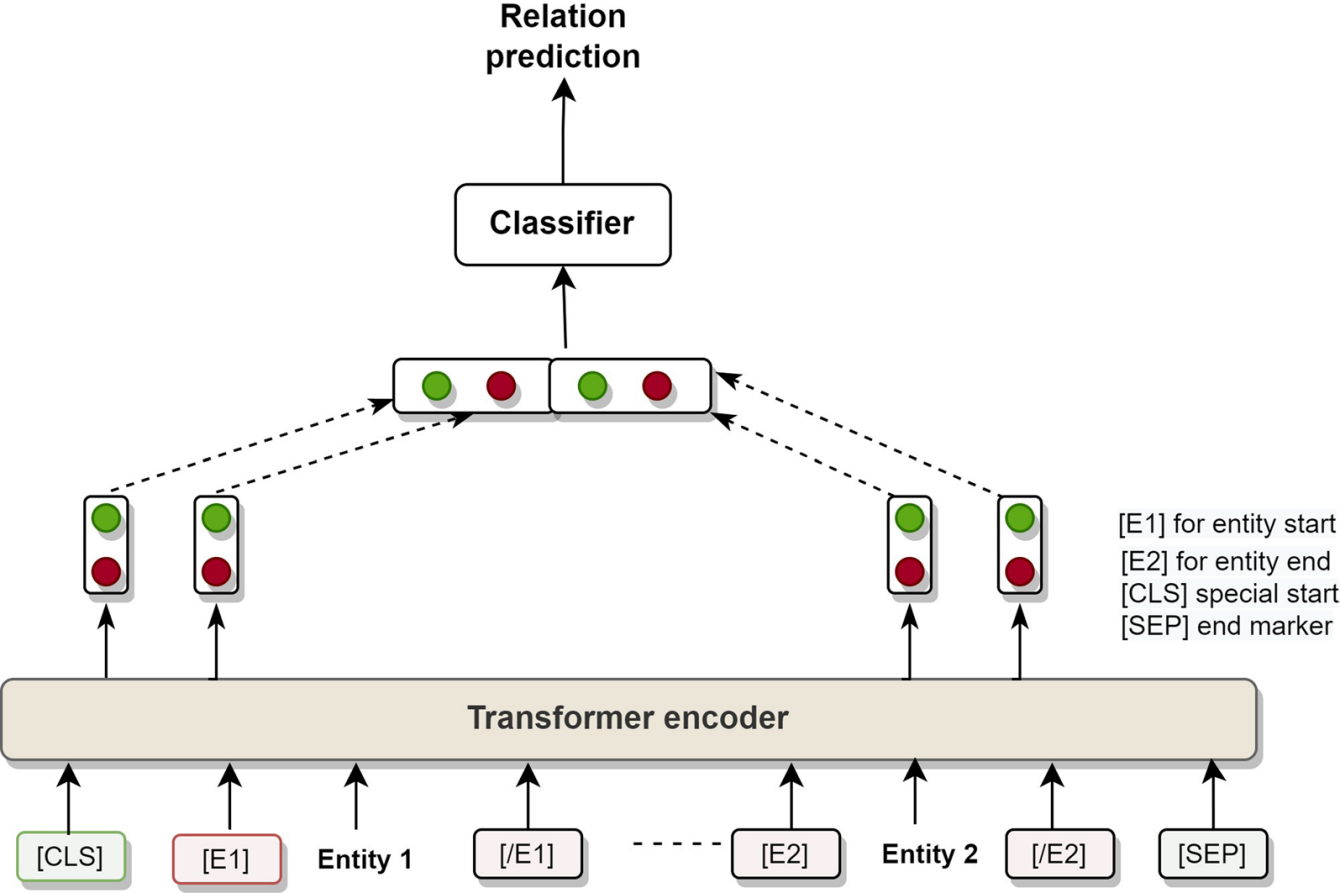
Zero-shot learning-based transformer model for relation extraction

Figure 3 shows the working of our ZSL-based Transformer model for Relation Extraction (ZSL-TRE). Our ZSL-TRE consists of a BERT encoder (Transformer layer) and a classifier layer, as shown in Fig. 3. The BERT encoder takes an input sequence of text and produces a fixed-length encoding that captures the contextual information in the input. This encoding is then passed to the classifier layer, which uses it to predict the relation expressed by the input. We use the softmax function in the classifier layer.

The BERT encoder is already trained on a large dataset of input–output pairs where the inputs are text sequences, and the outputs are class labels for a set of predefined relations. The trained model can classify a new input as expressing one of these relations, even if it has not seen that specific relation before, as long as the input is similar enough to the examples the model was trained on. The classifier layer is then trained to predict the relation based on the output of the BERT encoder, which encodes contextual information about the input. When presented with a new input, the BERT encoder encodes it and passes the encoding to the classifier, which then predicts the most likely relation based on the information it has learned from the training data. We show an example of RE from our corpus in Additional file 1: Figure S6.

#### Evaluation layer

The evaluation layer receives the NLP layer’s output (named entities and relations) and evaluates the results of the proposed NER and RE methods. This research uses a two-fold evaluation approach: quantitative analysis and qualitative analysis. We compare the proposed tasks’ accuracy to baseline methods across benchmark datasets (including our test set for NER) for the quantitative analysis. We also perform the ablation study to show the effectiveness of each part of our proposed model. To show how effective our proposed approach is for pandemic surveillance, we carried out a qualitative analysis using case report data. We demonstrate the use of different named entities and specify the unseen relations on the run. We show the summary of some data statistics of our reference dataset in Additional file 1: Table S6.

*Evaluation protocol* We randomly split each dataset into 70% train, 15% validation, and 15% test set for evaluation. We used 30% of our annotated data for the NER task test set. For the RE task, we used benchmark datasets as we don't have a test set. The details of the benchmark datasets and the baseline methods used in the evaluation are given in Additional file 1: Table S7. Similar to related works [12], we also employ the micro-average F1-score metric to evaluate NER and RE tasks. All the baselines are optimized to their optimal settings, and we report the best results for each method. The BERT encoder layers are implemented using the PyTorch BERT implementation available from Huggingface [70]. The general hyperparameters used during training are given in Additional file 1: Table S8.

*Training setup* We set the experimental environment as: Intel(R) Core(TM) i7-8565U CPU @ 1.80 GHz, 1.99 GHz, 16.0 GB RAM, 64-bit operating system, × 64-based processor; GPU: Google Colab Pro with cloud-based GPUs (K80, P100, or T4), 32 GB RAM and training capabilities. We connect the Google Colab to Google Drive to get enough storage for transfer learning.

## Results

### Overall performance comparison

The NER model performance is compared against the baseline methods using different benchmark datasets, including our own test set.

Our proposed method outperforms all the baseline methods on all of the datasets in Table 2. We observe that the BERT-based methods generally perform better than the BiLSTM-based methods, but the performance difference between the two sets of methods is relatively small, indicating that BiLSTM models can achieve a high level of accuracy if trained properly. We adopt a hybrid approach where we use the BERT-based model with the BiLSTM. Our approach to NER achieves a higher F1 score of 90.58% on the NCBI-disease dataset with significantly less feature engineering. On the BC5CDR dataset, our method obtains a micro-averaged F1 score of 89.90% for both chemical and disease entities. On the BC4CHEMD dataset with chemical entities, BERT-based methods, our method and Att-BiLSTM-CRF achieve scores above 90%. When evaluating the performance on the BC2GM and JNLPBA datasets for protein and gene names, our approach and the BERT-based methods perform well, with the overall performance on the JNLPBA dataset being relatively lower. This trend (of the lower performance of JNLPBA) is also observed in most related works [13] for these datasets. For the i2b2 datasets that are trained on clinical named entities, we find that clinical embeddings like those provided by BioBERT significantly improve the performance of clinical NER tasks, suggesting that a method performance is closely tied to the entity types it was trained on.

**Table 2:** Test results in biomedical NER task using the micro-averaged F1-score

Model/Dataset	NCBI	BC5 CDR	BC4 CHEMD	BC2 GM	JNL PBA	i2b2-clinical	i2b2-2012	Our data
*BiLSTM-based*								
BiLSTM-CRF [63]	85.80	85.92	89.48	80.60	74.29	85.66	86.10	88.10
BILSTM-CNN-Char [71]	*89.73*	87.73	88.06	87.51	77.29	84.08	83.10	89.20
BiLSTM-CRF-MTL [72]	88.85	84.93	89.42	82.12	77.03	83.25	83.24	87.10
Att-BiLSTM-CRF [73]	84.50	*88.93*	91.08	84.37	77.10	88.60	85.83	88.10
Doc-Att-BiLSTM-CRF [16]	88.60	87.30	85.10	81.80	76.23	85.18	85.17	86.94
BiLSTM-contextualized [74]	85.17	87.83	87.12	80.51	73.52	85.26	84.22	85.18
CollaboNet [75]	84.08	84.08	87.12	79.73	78.58	85.61	84.29	86.70
*BERT-based*								
SciBERT [76]	86.88	87.94	88.06	84.08	75.77	79.19	79.10	81.95
BLUE [77]	86.37	86.60	90.19	80.93	75.27	84.06	83.45	89.95
BioBERT-Base v1.0 [13]	87.71	85.80	90.77	84.72	77.59	85.64	86.00	90.10
BioBERT-Base v1.1 [13]	88.30	84.67	90.78	88.41	77.21	86.73	86.23	90.39
BioBERT-Base v1.2 [13]	88.15	86.70	**92.20**	*88.72*	*79.10*	*88.95*	*87.40*	*92.34*
Our approach	**90.58**	**89.90**	*91.58*	**89.15**	**79.92**	**89.10**	**90.10**	**94.78**

Bold means best score, italic second-best score

In the following experiments, we compare the performance of our proposed RE model with that of baseline methods using benchmark datasets. Since we do not have a specific labeled test set for the RE task, we evaluate the performance of our RE model on benchmark datasets and provide a detailed analysis of our approach on case reports in later sections. Our RE model utilizes a ZSL approach, in which we do not utilize the provided relation labels from the benchmark datasets. Instead, we attempt to infer these relations using the knowledge from our fine-tuned Transformer model. The baseline methods, on the other hand, are run using the relation labels provided by the benchmark dataset providers.

As shown in Table 3, our approach outperforms the other methods on all seven datasets. These other models employ various techniques to extract relationships from the input sentences and make predictions, and they have achieved strong performance with full supervision. However, our model can predict these relationships, which it has not seen before, with a higher level of accuracy. The superior performance of our method is attributed to its use of a transfer learning mechanism, where the relationship attributes are generated using zero-shot learning predictions.

**Table 3:** Test results of relation extraction task on benchmark datasets using micro-averaged F1-score

Method/Dataset	ADE	BioInfer	i2b2-clinical	i2b2-2012	CHEMPROT	JNLPBA	N2C2
C4.5 DT [35]	71.30	64.17	69.61	69.32	77.24	67.97	65.59
BiLSTM-CRF [36]	80.13	82.10	76.93	73.60	78.14	76.09	71.35
BiLSTM-CNN [78]	79.74	74.40	68.91	72.13	73.44	72.41	70.10
RNNs [36]	80.10	82.20	80.15	78.04	77.00	78.14	75.57
CMAN [37]	81.10	73.10	68.44	74.32	73.70	66.65	68.50
Adversial [37]	75.50	79.60	69.19	78.10	72.94	69.34	69.57
Multi-att-CNN [79]	79.24	84.35	70.43	78.80	77.50	71.62	70.15
BLUE BERT [77]	80.39	82.09	72.32	79.10	79.98	80.56	79.10
BioBERT [13]	*82.03*	*86.90*	*81.20*	*80.23*	*81.46*	*83.41*	*79.18*
Our approach	**90.00**	**88.88**	**90.12**	**85.03**	**88.95**	**84.50**	**84.10**

Bold means best score, italic second-best score

### Ablation study

To verify the validity of each part of our proposed NER and RE models, we conducted the ablation experiment. To keep it concise and avoid presenting repetitive test results, we have focused the ablation experiment on the i2b2-clinical and our own test set. The ablation experiment settings are as follows:

- *Full*: Proposed Transformer-BiLSTM-CRF model.
- *BiLSTM-CRF*: Remove the Transformer layer. Equivalent to BiLSTM-CRF [63] with character-level embedding via the CNN layer.
- *BiLSTM:* Remove the Transformer and CRF layers, equivalent to the BiLSTM model with a softmax layer to predict NER labels.
- *Transformer-BiLSTM*: Remove the CRF layer only, a softMax layer to predict NER labels.
- *Transformer:* Remove the BiLSTM and CRF layers.

According to the results presented in Table 4, the full proposed model has the highest micro-average F1 score among the model variants on both test sets. The BiLSTM-CRF model can capture global features, but its performance is decreased by 4–6% compared to the full model, probably because it lacks contextualized representations by missing Transformer input. The model variants with a Transformer layer outperform those without it. The CRF layer, which is added after the BiLSTM and is used to correct the named entity tag sequence, is not present in the Transformer variants. Despite this, the Transformer variants still perform well with a simple enhancement function applied to the IOB representation of the output. Overall, this result suggests that transfer learning has improved the performance of the model variants.

**Table 4:** Ablation experiment results of the proposed NER model on i2b2-clinical and our test set using micro-averaged F1-score

Model	i2b2-clinical	Our data
Full	**90.10**	**94.78**
BiLSTM-CRF	86.10	88.10
BiLSTM	85.13	87.63
Transformer-BiLSTM	*88.12*	*90.28*
Transformer	87.10	90.23

Bold means best score, italic second-best score

We again perform the ablation experiment on the proposed RE model. We present the results for the i2b2-clinical dataset in this report, as we do not have our test set for this task. The model variants are:

- *Full*: Proposed RE model without any labeled data, and task-specific model weights.
- *Without fine-tuning step*: BERT-based layer without a task-specific model.
- *Without full Transformer layer*: BILSTM-CRF model without the Transformer layer.

Overall, the results in Table 5 show that the full model with task-specific fine-tuning performed the best. This is because the fine-tuning process adjusts the model weights to be more suitable for the specific task, leading to better performance. When the fine-tuning step is skipped and only the Transformer layer is retained, the model is not as effective at the task, resulting in lower performance. When the full transformer layer is missing, the model performs even worse, likely because the model is unable to predict the relations in the test set effectively.

**Table 5:** Ablation experiment results of the proposed NER model using micro-averaged F1-score on the relations provided in the i2b2-clinical testset

Model	i2b2-clinical
Full: proposed RE model	**90.12**
Without fine-tuning step	82.35
Without a full Transformer layer	78.9

Bold means best score

### Evaluation outcomes

#### Effectiveness of named entity recognition approach

We evaluated the effectiveness of our proposed approach in practical use cases.

**Fig. 4:**
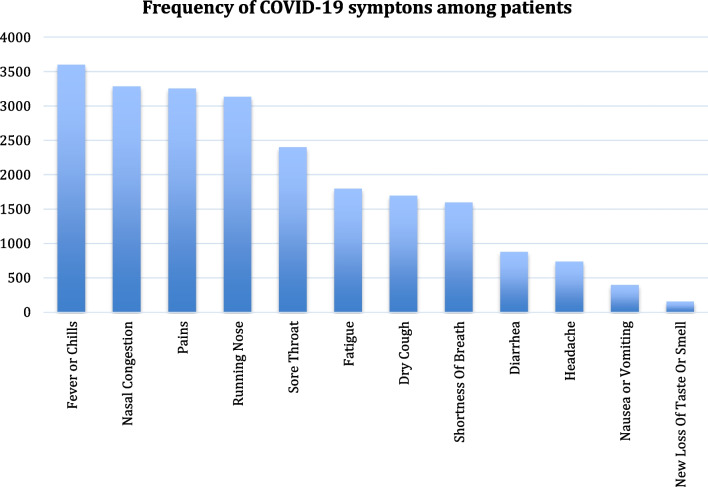
Frequency of COVID-19 symptoms

**Fig. 5:**
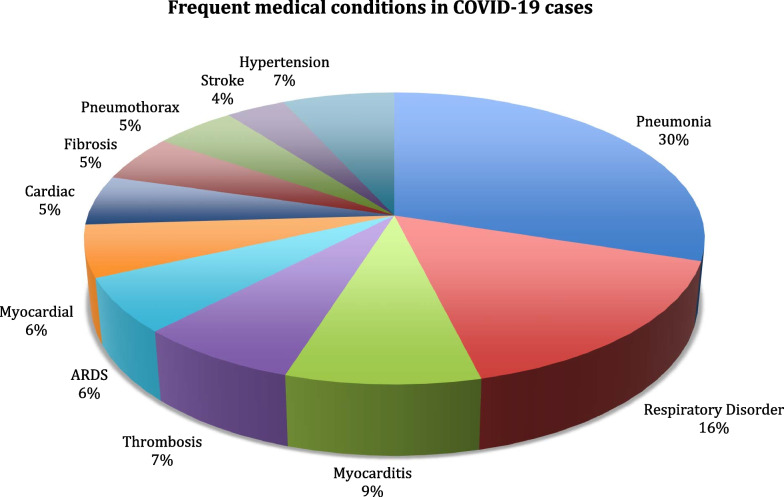
Distribution of most frequent medical complications in the population

We observe in Fig. 4 that fever/chills, nasal congestion, pains, and running nose are the most frequent COVID-19 symptoms, which are reported by the CDC [80]. Next, we show the most frequent medical conditions in COVID-19 patients in Fig. 5 and find pneumonia and respiratory disorders as the most frequent among others.

**Fig. 6:**
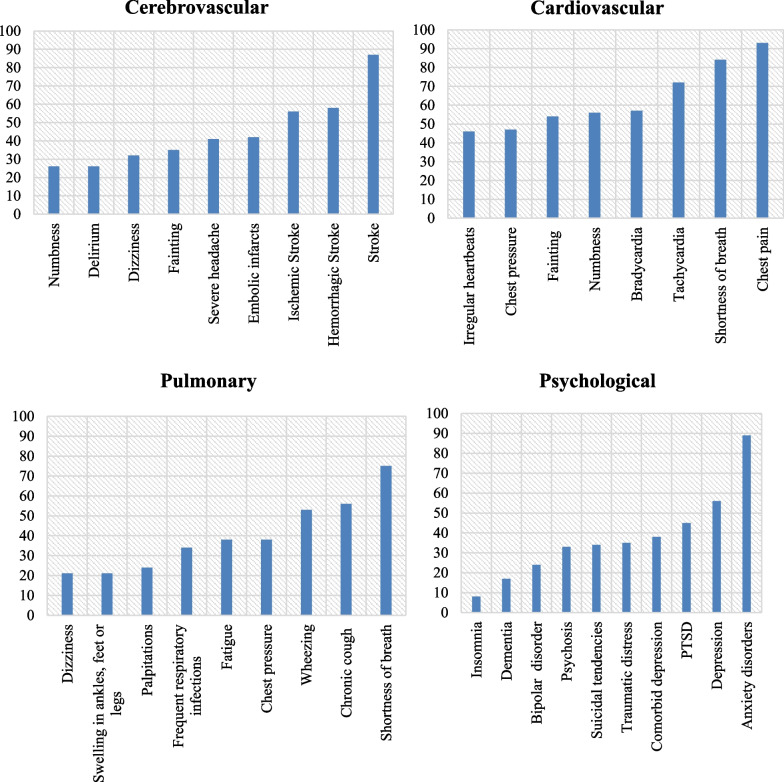
Condition prevalence related to different disease syndromes (Cerebrovascular, Cardiovascular, Pulmonary, Psychological). Bars represent the number of respondents who experienced each symptom at any point in their illness

We also show the prevalence of conditions in COVID-19 patients based on the most occurring disease disorder (occurring more than 70%). The results are shown in Fig. 6.

According to Fig. 6, stroke appears to be the most common condition among patients with the cerebrovascular syndrome, chest pain is the most common condition among those with cardiovascular disease, and shortness of breath is the most prevalent condition among those with pulmonary disease. These findings highlight the serious nature of these conditions among patients with COVID-19. Additionally, we see that psychological conditions such as anxiety, depression, and post-traumatic stress disorder (PTSD) are present in COVID-19 patients, which may be the result of the impact of COVID-19 on their mental health.

**Fig. 7:**
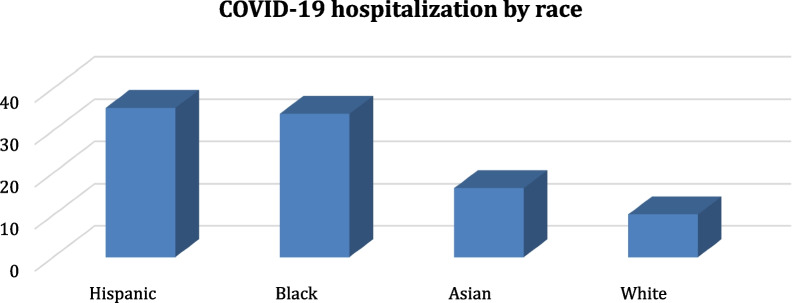
COVID-19 hospitalization by race

Figure 7 depicts COVID-19 hospitalization by race, revealing that Hispanics are the most affected (37%), followed by blacks (35%), Asians (17%), and whites (11%). This finding is based on a sample of the population and is not representative of the whole population.

In Additional file 1: Table S9 we show that named entities occur frequently in 1000 random case reports. We also show the hospitalization, ICU admission, and mortality in COVID-19 patients of various ages in Additional file 1: Figure S7.

#### Effectiveness of relation extraction approach

We demonstrate the effectiveness of our ZSL-TRE approach by specifying relationships on the run. Table 6 displays the 'after' relationships—condition/symptom followed by a disease disorder.

**Table 6:** ‘After’ relation—disease disorder—condition/symptom

Disease disorder	Following conditions/symptoms
COVID-19	Fever, cough, respiratory symptoms, anosmia, ageusia, tachypneic, long COVID, acute respiratory failure, extensive pulmonary fibrosis, high-grade fever, severe gastrointestinal
Coronary artery disease	Hypertension, dyslipidemia
Acute kidney injury	Hyperkalemia, severe metabolic acidosis, hyperlactatemia
Acute respiratory failure	Extensive pulmonary fibrosis, mixed venous oxygen saturation, SARS-CoV-2
Chest pain	Myalgia, headache, pressure, palpitations, shortness of breath
Dry cough	Rhinorrhea, nausea, vomiting
Episodic shortness of breath	Nocturnal tachycardia, chest pain, nocturnal tachycardia, chest pain
Hypertension	Shortness of breath, cough, gout, heart failure, reduced ejection fraction, chronic kidney disease, asthenia, weight loss, anosmia, ageusia

Disease disorders are chosen based on the frequency of prevalence (occurring > 70%)

**Fig. 8:**
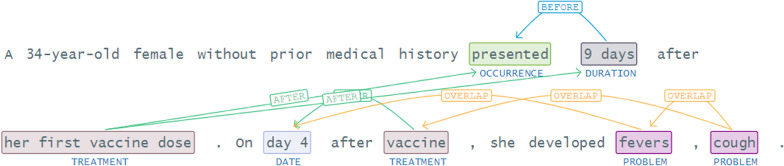
Temporal relations in a text

Table 6 shows that certain symptoms are followed by a specific disease; for example, the symptoms of COVID-19 are visible once a patient has the disease. We also specify the temporal relation: before, after, and overlap relations [81], as defined in the 2012 i2b2 challenge in Fig. 8. We observe that conditions (fever and cough) are seen after the vaccine (treatment).

Next, we specify the relationship between “DRUG AND [EFFECT]” and show the top 3 effects for a commonly mentioned drug (Oral amoxicillin) in Table 7. The result shows that abdominal pain and diarrhea are some of the effects associated with amoxicillin and Pirfenidone.

**Table 7:** Relations type: DRUG -EFFECT

Drug	Effect	Drug	Effect	Drug	Effect
Oral amoxicillin	Abdominal pain, loose stools, worsening rash, new-onset painful joint swelling	Pirfenidone	Skin rash, swelling, ulcer, diarrhea	BNT162b2 vaccine	Acute headache, fever, nausea, vomiting, oral aphthous, ulcers

**Fig. 9:**
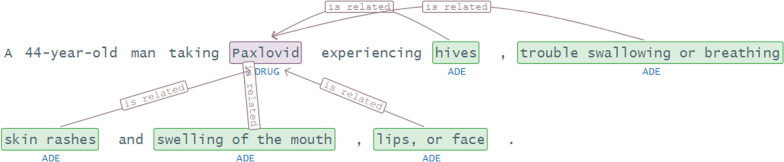
Adverse drug effect with Paxlovid drug

We also show the adverse drug effect (ADE) associated with the Paxlovid drug, which is most frequently for treating COVID-19, in Fig. 9 and see the associated effects with this drug are hives, trouble breathing, skin and swelling.

## Discussion

### Principal findings

We have observed that pneumonia, respiratory infections, and acute respiratory distress syndrome (ARDS) are common symptoms among COVID-19 patients, which is consistent with the reports from the WHO [82]. We have also noted the prevalence of various conditions among patients with multiple comorbidities and how different symptoms and conditions become more prominent in these patients. Our findings on psychological conditions and their potential relationship with Long-COVID and mental health sequelae [83] may be useful for practitioners to consider when treating patients. We have also identified relationships between certain drugs and side effects such as headache, nausea, and dizziness, which can help healthcare providers quickly identify potential adverse effects in patients without having to manually review EHRs.

### Impact of transfer learning for predicting COVID-19 patients’ outcomes

In the context of detecting COVID-19 named entities and relationships, transfer learning can be used to leverage existing models and knowledge about NLP to improve the performance of a new model on a specific task. This can be particularly useful when there is a limited amount of annotated data available for the specific task, and the model can use the knowledge acquired from other tasks to better understand the data and make more accurate predictions. In our experiments, we found that our methods, BioBERT, and BLUE, which all use transfer learning, performed very well in detecting named entities and relationships, suggesting that this approach can be effective in this domain (addressing RQ stated above).

### Generalizability of the proposed approach

Our proposed framework has the potential to be applied to other domains and tasks with some adjustments to the data and possibly minor code modifications. The extent to which this framework can be generalized to other domains and tasks depends on the specific characteristics of the domain and task at hand. Some adjustments will likely need to be made to the data and possibly to the code to apply the framework to other domains and tasks.

### Active learning experience

In our study, we discovered that active learning has the potential to reduce annotation costs for building NER models. Our current experience with active learning is based on a simple use case, but the overall goal with this method was to show that it is worth considering, particularly in applications where the data domain is limited (such as COVID-19 or a specific use case). We suggest further investigation into different active learning techniques for large-scale re-annotation.

### Predicting unseen relations in the texts using NLP

In this work, we attempted to infer relationships in text using NLP techniques. While this approach allows us to identify relationships between entities, it is not the same as the causal relationship extraction task that is commonly used in epidemiological studies. Most existing relationship extraction techniques in ML require labeled data for supervised learning tasks, and this can be a significant challenge. However, our approach does not require labeled data, making it a potential alternative for extracting relationships in the texts.

### Limitations and ethical implications of associations and relations with NLP extraction tools

NLP techniques are often used to extract and analyze relationships and associations from text data [23, 84], but like any analytical method, they have limitations and potential biases that should be carefully considered. Association analysis can be a useful tool for identifying patterns and relationships in data [85], but it is important to recognize that the presence of an association does not necessarily indicate a causal relationship. There may be other factors at play that contribute to the observed association, and it is important to consider those factors when interpreting the results. For example, in the case of hospitalization by race discussed above, there may be other factors that contribute to the association between race and hospitalization [86], such as socio-economic status, access to healthcare, or pre-existing health conditions. If these factors are not taken into account, the analysis of the data could be misleading and potentially perpetuate harmful stereotypes or biases. It is important for researchers and analysts to be mindful of these limitations in text-based analysis and to consider potential alternative explanations for observed associations.

The source data we used is focused on published case reports. As a result, the sample is likely biased toward sicker patients, those hospitalized, those who had Long-COVID, and those who were seen by academic physicians who would write them up for publication. It excludes minor cases, those who were not hospitalized, and those who were not cared for by these providers, who were likely poorer, lived-in remote areas, did not receive proper care and were less likely to see academic physicians so on.

Deploying a language model on a large dataset, particularly in the clinical text domain, requires powerful computing resources to process and analyze the data efficiently. Insufficient hardware such as lack of memory and graphics processing units can impede the speed and accuracy of the analysis and decision-making process. Furthermore, it is vital to ensure patient privacy and comply with regulatory requirements, such as the Health Insurance Portability and Accountability Act (HIPAA) [87], when working with clinical text. Therefore, it is essential to include measures such as implementing secure protocols and ensuring compliance with Protected Health Information (PHI) regulations throughout the NLP pipeline to ensure the success of the deployment.

### Future directions

We propose leveraging the Bradford Hill criteria [88] in the RE task. The Bradford Hill criteria are a tool that can be used to assess the causal relationship between an exposure and an outcome, and by leveraging these criteria and coordinating PH initiatives with the RE task, it may be possible to identify and address potential health risks and issues within a population more effectively.

The integration of additional data sources, such as real-time EHRs and pathologic reports, is important in effectively utilizing AI in the fight against the COVID-19 pandemic. Not only will this provide a more comprehensive understanding of the disease, but it will also aid in the development of more accurate and effective treatment plans. Furthermore, it is essential that we address specific aspects of the pandemic, such as misinformation spread, as this can greatly impact the effectiveness of our efforts.

In terms of Inter-Annotator Agreement (IAA), it is imperative that we utilize additional statistical measures [55], such as Cohen Kappa, Fleiss Kappa, and Krippendorff Alpha. These measures provide a more in-depth understanding of the reliability and consistency of annotations made by different annotators and can help identify any areas of disagreement or confusion that may require attention. By using various measures, we can ensure the highest level of accuracy and precision in our AI-assisted efforts to combat the pandemic.

## Conclusion

This study has shown that NLP-based methods can be used to detect the presence of diseases, symptoms, and risk characteristics. Transfer learning shows promise for developing predictive disease models with limited data, and our proposed methodology offers a useful way to identify named entities and relationships in clinical texts. In comparison to state-of-the-art methods, our proposed methods achieve a higher micro-averaged F1 score for both the NER and RE tasks. The analysis of the case report data shows that the proposed approach can be an effective tool for pandemic surveillance. Overall, this study demonstrates the potential of NLP-based methods for detecting and understanding diseases and other clinical concepts in text data.

## Supplementary Information

**Additional file 1: Table S1**. Search query for data cohort. **Table S2.** Named entities. **Fig. S1.** Active learning for data annotation. **Fig. S2.** Task-specific Transformer model for named entities task. **Table S3.** Notations used in the paper. **Fig. S3.** IOB format by CRF layer. **Table S4.** Case study: Named entities extracted from the case report (case report text only). **Fig. S4.** Case study, Visual representation of named entities from the snippet of case report. **Table S5.** NER on a general case report [3]. **Fig. S5.** Dependency parsing. Figure S6: Relation between disease disorder (entity) and psychological condition (entity). **Table S6.** Natural language processing-based summary of COVID-19 cohort. **Table S7.** Benchmark datasets and methods. **Table S8.** Hyperparameter and best result value (values in parenthesis represent the parameter ranges tested). **Table S9.** High frequency named entities in case reports. **Fig. S7.** Hospitalization, ICU admission, and morality in COVID-19 patients with different age groups

## Data Availability

The data underlying this article will be shared on reasonable request to the corresponding author.

## Abbreviations

COVID-19: Coronavirus disease
PH: Public health
SDOH: Social determinants of health
EHR: Electronic health records
WHO: World Health Organization
NLP: Natural language processing
ML: Machine learning
IAA: Inter-annotator agreement
NER: Named entity recognition
RE: Relation extraction
NIH: National Institutes of Health
NLM: National Library of Medicine
CARE: Case reports
IOB: Inside–outside–before
BERT: Bidirectional encoder representations from transformers
BioBERT: Bidirectional encoder representations from transformers for biomedical text mining
BiLSTM: Bidirectional long short-term memory
CRF: Conditional random field
CoNLL: Conference on computational natural language learning
DP: Dependency parsing
ZSL: Zero shot learning
ZSL-TRE: ZSL-based transformer for relation extraction
NCBI: National center for biotechnology information
BC5CDR: Biocreative v chemical disease relations
BC4CHEMD: Biocreative iv chemical and drug
BC2GM: Biocreative ii gene mention recognition
I2B2: Informatics for integrating biology and the bedside
ADE: Adverse drug events
BioInfer: Bio information extraction resource
CHEMPROT: Chemical-protein interactions
N2C2: National NLP clinical challenges
CNN: Convolutional neural network
MTL: Multi-task learning
Att: Attention
Doc: Document
CollaboNet: Collaboration of deep neural networks
SciBERT: Science BERT
C4.5 DT: C45 decision tree
RNN: Recurrent neural network
CMAN: Cross-Modal Attention Network
ADV: Adversial
ARDS: Acute respiratory distress syndrome
PTSD: Post-traumatic stress disorder
ME/CFS: Myalgic encephalomyelitis/chronic fatigue syndrome

## References

[1] Ourworldindata.org. COVID-19 Data Explorer. Our world in data. 2022.

[2] Flor LS, Friedman J, Spencer CN, Cagney J, Arrieta A, Herbert ME, et al. Quantifying the effects of the COVID-19 pandemic on gender equality on health, social, and economic indicators: a comprehensive review of data from March, 2020, to September, 2021. Lancet. 2022. 10.1016/S0140-6736(22)00008-3 PMC8890763 35247311

[3] Baena-Diéz JM, Barroso M, Cordeiro-Coelho SI, Diáz JL, Grau M (2020). Impact of COVID-19 outbreak by income: hitting hardest the most deprived. J Public Heal.

[4] Kaye AD, Okeagu CN, Pham AD, Silva RA, Hurley JJ, Arron BL (2021). Economic impact of COVID-19 pandemic on healthcare facilities and systems: International perspectives. Best Pract Res Clin Anaesthesiol.

[5] Williamson EJ, Walker AJ, Bhaskaran K, Bacon S, Bates C, Morton CE (2020). Factors associated with COVID-19-related death using OpenSAFELY. Nature.

[6] Caufield JH, Zhou Y, Bai Y, Liem DA, Garlid AO, Chang K-W, et al. A comprehensive typing system for information extraction from clinical narratives. medRxiv. 2019;19009118.

[7] Raza S, Schwartz B. Detecting biomedical named entities in COVID-19 texts. In: Workshop on healthcare AI and COVID-19, ICML 2022; 2022.

[8] Weiss K, Khoshgoftaar TM, Wang D (2016). A survey of transfer learning. J Big Data.

[9] Settles B (2010). Active learning literature survey. Mach Learn.

[10] Nadeau D, Sekine S (2007). A survey of named entity recognition and classification. Lingvisticae Investig.

[11] Campos D, Matos S, Oliveira JL (2012). Biomedical named entity recognition: a survey of machine-learning tools. Theory Appl Adv Text Min.

[12] Cho H, Lee H (2019). Biomedical named entity recognition using deep neural networks with contextual information. BMC Bioinform.

[13] Lee J, Yoon W, Kim S, Kim D, Kim S, So CH (2020). BioBERT: a pre-trained biomedical language representation model for biomedical text mining. Bioinformatics.

[14] Alsentzer E, Murphy JR, Boag W, Weng W-H, Jin D, Naumann T, et al. Publicly available clinical BERT embeddings. Preprint http://arxiv.org/abs/190403323. 2019.

[15] Raza S, Schwartz B, Rosella LC (2022). CoQUAD: a COVID-19 question answering dataset system, facilitating research, benchmarking, and practice. BMC Bioinform.

[16] Xu K, Yang Z, Kang P, Wang Q, Liu W (2019). Document-level attention-based BiLSTM-CRF incorporating disease dictionary for disease named entity recognition. Comput Biol Med.

[17] Gao S, Kotevska O, Sorokine A, Christian JB (2021). A pre-training and self-training approach for biomedical named entity recognition. PLoS ONE.

[18] Wu C, Luo G, Guo C, Ren Y, Zheng A, Yang C (2020). An attention-based multi-task model for named entity recognition and intent analysis of Chinese online medical questions. J Biomed Inform.

[19] Crichton G, Pyysalo S, Chiu B, Korhonen A (2017). A neural network multi-task learning approach to biomedical named entity recognition. BMC Bioinform.

[20] Du X, Kang K, Chong Y, Zhang ML, Yang W, Meng XL (2021). COVID-19 patient with an incubation period of 27 d: a case report. World J Clin Cases.

[21] Kumar S. A survey of deep learning methods for relation extraction. Preprint http://arxiv.org/abs/170503645. 2017.

[22] Zhou D, Zhong D, He Y. Biomedical relation extraction: from binary to complex. Comput Math Methods Med. 2014;2014. 10.1155/2014/298473 PMC4156999 25214883

[23] Yang J, Han SC, Poon J (2022). A survey on extraction of causal relations from natural language text. Knowl Inf Syst.

[24] Zeng D, Liu K, Lai S, Zhou G, Zhao J. Relation classification via convolutional deep neural network. In: Proceedings of COLING 2014, the 25th international conference on computational linguistics: technical papers, 2014. p. 2335–44.

[25] Miwa M, Bansal M. End-to-end relation extraction using lstms on sequences and tree structures. Preprint http://arxiv.org/abs/160100770. 2016.

[26] Pushp PK, Srivastava MM. Train once, test anywhere: zero-shot learning for text classification. Preprint http://arxiv.org/abs/171205972. 2017.

[27] Levy O, Seo M, Choi E, Zettlemoyer L. Zero-shot relation extraction via reading comprehension. Preprint http://arxiv.org/abs/170604115. 2017.

[28] Obamuyide A, Vlachos A. Zero-shot relation classification as textual entailment. In: Proceedings of the first workshop on fact extraction and VERification (FEVER). 2018. p. 72–8.

[29] Chen C-Y, Li C-T. ZS-BERT: Towards zero-shot relation extraction with attribute representation learning. In: Toutanova K, Rumshisky A, Zettlemoyer L, Hakkani-Tür D, Beltagy I, Bethard S, et al., editors. Proceedings of the 2021 Conference of the North American Chapter of the Association for Computational Linguistics: Human Language Technologies, {NAACL-HLT} 2021, Online, June 6–11, 2021. Association for Computational Linguistics; 2021. p. 3470–9.

[30] Devlin J, Chang MW, Lee K, Toutanova K. BERT: Pre-training of deep bidirectional transformers for language understanding. Preprint http://arxiv.org/abs/181004805. 2018.

[31] Lybarger K, Ostendorf M, Thompson M, Yetisgen M (2021). Extracting COVID-19 diagnoses and symptoms from clinical text: a new annotated corpus and neural event extraction framework. J Biomed Inform.

[32] Luo X, Gandhi P, Storey S, Huang K (2021). A deep language model for symptom extraction from clinical text and its application to extract covid-19 symptoms from social media. IEEE J Biomed Heal Inform.

[33] Lu Wang L, Lo K, Chandrasekhar Y, Reas R, Yang J, Eide D, et al. CORD-19: the Covid-19 open research dataset. 2020.

[34] Silverman GM, Sahoo HS, Ingraham NE, Lupei M, Puskarich MA, Usher M (2021). Nlp methods for extraction of symptoms from unstructured data for use in prognostic covid-19 analytic models. J Artif Intell Res.

[35] Girju R. Automatic detection of causal relations for question answering. 2003;76–83.

[36] Hsieh Y-L, Chang Y-C, Chang N-W, Hsu W-L. Identifying protein-protein interactions in biomedical literature using recurrent neural networks with long short-term memory. In: Proceedings of the eighth international joint conference on natural language processing (volume 2: short papers). 2017. pp. 240–5.

[37] Zhao S, Hu M, Cai Z, Liu F. Modeling dense cross-modal interactions for joint entity-relation extraction. In: Proceedings of the twenty-ninth international conference on international joint conferences on artificial intelligence. 2021. pp. 4032–8.

[38] Zhu Y, Li L, Lu H, Zhou A, Qin X (2020). Extracting drug-drug interactions from texts with BioBERT and multiple entity-aware attentions. J Biomed Inform.

[39] Lung J. Application of NLP to extract biomedical entities from COVID-19 papers. 2021.

[40] Liu Z, Yang M, Wang X, Chen Q, Tang B, Wang Z, et al. Entity recognition from clinical texts via recurrent neural network. 10.1186/s12911-017-0468-7. 10.1186/s12911-017-0468-7 PMC5506598 28699566

[41] Zhou Y, Ju C, Caufield JH, Shih K, Chen C, Sun Y, et al. Clinical named entity recognition using contextualized token representations. 2021.

[42] Perera N, Dehmer M, Emmert-Streib F (2020). Named entity recognition and relation detection for biomedical information extraction. Front Cell Dev Biol.

[43] Rison RA, Shepphird JK, Kidd MR (2017). How to choose the best journal for your case report. J Med Case Rep.

[44] National Center for Biotechnology Information. Definitions. 2020. https://www.ncbi.nlm.nih.gov.

[45] IMI. CARE case report guidelines. 2019.

[46] Nussbaumer-Streit B, Klerings I, Dobrescu AI, Persad E, Stevens A, Garritty C (2020). Excluding non-English publications from evidence-syntheses did not change conclusions: a meta-epidemiological study. J Clin Epidemiol.

[47] Spark OCR- John Snow Labs. 2022. https://nlp.johnsnowlabs.com/docs/en/ocr.

[48] Elasticsearch. 2014. https://www.elastic.co.

[49] Brady EL, Wallenstein MB (1967). The national standard reference data system. Science.

[50] Cardoso JR, Pereira LM, Iversen MD, Ramos AL (2014). What is gold standard and what is ground truth?. Dent Press J Orthod.

[51] Caufield JH. MACCROBAT. 2020. 10.6084/m9.figshare.9764942.v2.

[52] Annotation Lab - FREE by John Snow Labs. 2022.

[53] Doğan RI, Leaman R, Lu Z (2014). NCBI disease corpus: a resource for disease name recognition and concept normalization. J Biomed Inform.

[54] Nothman J, Ringland N, Radford W, Murphy T, Curran JR (2013). Learning multilingual named entity recognition from Wikipedia. Artif Intell.

[55] Artstein R. Inter-annotator agreement. In: Handbook of linguistic annotation. Springer; 2017. p. 297–313.

[56] Tjong Kim Sang EF, de Meulder F. Introduction to the CoNLL-2003 shared task: language-independent named entity recognition. In: Proc 7th Conf Nat Lang Learn CoNLL 2003 HLT-NAACL 2003; 2003. pp. 142–7.

[57] Chen Y, Lasko TA, Mei Q, Denny JC, Xu H (2015). A study of active learning methods for named entity recognition in clinical text. J Biomed Inform.

[58] Chaybouti S, Saghe A, Shabou A. EfficientQA : a RoBERTa based phrase-indexed question-answering system. 2021; figure 1:1–9.

[59] shainaraza. bner-biobert. GitHub. 2022.

[60] Huang Z, Xu W, Yu K. Bidirectional LSTM-CRF models for sequence tagging. 2015.

[61] Vaswani A, Shazeer N, Parmar N, Uszkoreit J, Jones L, Gomez AN, et al. Attention is all you need. In: Advances in neural information processing systems. 2017. p. 5998–6008.

[62] Hochreiter S, Schmidhuber J (1997). Long short-term memory. Neural Comput.

[63] Lample G, Ballesteros M, Subramanian S, Kawakami K, Dyer C. Neural architectures for named entity recognition. Preprint http://arxiv.org/abs/160301360. 2016.

[64] Lafferty J, Mccallum A, Pereira F. Conditional random fields : probabilistic models for segmenting and labeling sequence data abstract. 1999;2001:282–9

[65] Sexton T. IOB Format Intro—Nestor. 2022.

[66] Gilio L, Galifi G, Centonze D, Stampanoni-Bassi M (2022). Case Report: overlap between long COVID and functional neurological disorders. Front Neurol.

[67] El-naggar HA, El-Mahallawy YA, Harby MI, Abou Madawi NA (2023). Bilateral collagenous fibroma of the hard palate: a case report and review of the literature. J Med Case Rep.

[68] Nivre J, Scholz M. Deterministic dependency parsing of English text. In: COLING 2004: proceedings of the 20th international conference on computational linguistics. 2004. pp. 64–70.

[69] Tang R, Nogueira R, Zhang E, Gupta N, Cam P, Cho K, et al. Rapidly bootstrapping a question answering dataset for COVID-19. 2020. arxiv:2004.11339

[70] huggingface. transformers. GitHub. 2022.

[71] Chiu JPC, Nichols E (2016). Named entity recognition with bidirectional LSTM-CNNs. Trans Assoc Comput Linguist.

[72] Wang X, Zhang Y, Ren X, Zhang Y, Zitnik M, Shang J (2019). Cross-type biomedical named entity recognition with deep multi-task learning. Bioinformatics.

[73] Luo L, Yang Z, Yang P, Zhang Y, Wang L, Lin H (2018). An attention-based BiLSTM-CRF approach to document-level chemical named entity recognition. Bioinformatics.

[74] Akbik A, Blythe D, Vollgraf R. Contextual string embeddings for sequence labeling. IN: COLING 2018 - 27th Int Conf Comput Linguist Proc. 2018. pp. 1638–49.

[75] Yoon W, So CH, Lee J, Kang J (2019). Collabonet: collaboration of deep neural networks for biomedical named entity recognition. BMC Bioinform.

[76] Beltagy I, Lo K, Cohan A. SCIBERT: A pretrained language model for scientific text. In: EMNLP-IJCNLP 2019 - 2019 conference on empirical methods in natural language processing and 9th international joint conference on natural language processing, proceedings of the conference, 2020. pp. 3615–20.

[77] Peng Y, Yan S, Lu Z. Transfer learning in biomedical natural language processing: an evaluation of BERT and ELMo on ten benchmarking datasets. Preprint http://arxiv.org/abs/190605474. 2019.

[78] Quan C, Luo Z, Wang S (2020). A hybrid deep learning model for protein–protein interactions extraction from biomedical literature. Appl Sci.

[79] Wang L, Cao Z, De Melo G, Liu Z. Relation classification via multi-level attention cnns. In: Proceedings of the 54th annual meeting of the association for computational linguistics (Volume 1: Long Papers). 2016. pp. 1298–307.

[80] Singh J (2004). Centers for disease control and prevention. Indian J Pharmacol.

[81] Lee H-J, Zhang Y, Jiang M, Xu J, Tao C, Xu H (2018). Identifying direct temporal relations between time and events from clinical notes. BMC Med Inform Decis Mak.

[82] Egdahl A (1954). WHO: World Health Organization. Ill Med J.

[83] Akbarialiabad H, Taghrir MH, Abdollahi A, Ghahramani N, Kumar M, Paydar S (2021). Long COVID, a comprehensive systematic scoping review. Infection.

[84] Patra BG, Sharma MM, Vekaria V, Adekkanattu P, Patterson OV, Glicksberg B (2021). Extracting social determinants of health from electronic health records using natural language processing: a systematic review. J Am Med Inform Assoc.

[85] Tan P-N, Kumar V, Srivastava J (2004). Selecting the right objective measure for association analysis. Inf Syst.

[86] Rutherford A. How to argue with a racist: History, science, race and reality. UK: Hachette; 2020.

[87] (OCR) O for CR. Methods for de-identification of PHI. HHS.gov. 2012.

[88] Rothman KJ, Greenland S (2005). Hill’s criteria for causality. Encycl Biostat.

