# Supplementary material for: Entity and relation extraction from clinical case reports of COVID-19: a natural language processing approach
Source: BMC Med Inform Decis Mak. 2023 Jan 26;23:20. doi: 10.1186/s12911-023-02117-3 (PMC9879259; doi:10.1186/s12911-023-02117-3)
Supplement: Supplementary file 1 — Additional file 1: Table S1. Search query for data cohort. Table S2. Named entities. Fig. S1. Active learning for data annotation. Fig. S2. Task-specific Transformer model for named entities task. Table S3. Notations used in the paper. Fig. S3. IOB format by CRF layer. Table S4. Case study: Named entities extracted from the case report (case report text only). Fig. S4. Case study, Visual representation of named entities from the snippet of case report. Table S5. NER on a general case report [3]. Fig. S5. Dependency parsing. Figure S6: Relation between disease disorder (entity) and psychological condition (entity). Table S6. Natural language processing-based summary of COVID-19 cohort. Table S7. Benchmark datasets and methods. Table S8. Hyperparameter and best result value (values in parenthesis represent the parameter ranges tested). Table S9. High frequency named entities in case reports. Fig. S7. Hospitalization, ICU admission, and morality in COVID-19 patients with different age groups [file 12911_2023_2117_MOESM1_ESM.docx]

**Benchmarking a Data Cohort Using Literature of Reported Cases: A Novel Natural Language Processing Approach**

Shaina Raza ^1,2*,^ PhD; Brian Schwartz ^1,2^, MD, MScCH

^1^ Public Health Ontario (PHO),

Toronto, ON, Canada.

^2^ Dalla Lana School of Public Health, University of Toronto,

Toronto, ON, Canada.

Corresponding Author: [shaina.raza@oahpp.ca](mailto:shaina.raza@oahpp.ca)

**Appendix A: Analysis**

**Table S1:** Search query for data cohort

| **Search:** COVID AND LONG COVID  Filters: Free full text, Case Reports, English, Child: 6-12 years, Adolescent: 13-18 years, Adult: 19-44 years, Middle Aged: 45-64 years, Aged: 65+ years.  COVID*:* "sars-cov-2"OR "sars-cov-2" OR "covid" OR "covid-19"OR "covid-19"  LONG COVID*:* "post-acute COVID-19 syndrome" OR "post-acute COVID-19 syndrome" OR "long covid"  *Query*: (("sars cov 2" OR "sars cov 2" OR "covid" OR "covid 19"OR "covid 19") AND ("post acute covid 19 syndrome"OR "post acute covid 19 syndrome" OR "long covid")) AND (case reports) AND (english) AND (child OR adolescent OR adult OR middle aged OR aged)) |
| --- |

**Table S2:** Named entities

| Named entities | | |
| --- | --- | --- |
| GENDER  HEIGHT  AGE  DATE  WEIGHT  SMOKING  RACE  RELATIVE_DATE  DRUG_NAME  DURATION  ADMISSION_DISCHARGE  ALCOHOL  SUBSTANCE  EMPLOYMENT  TIME | OXYGEN_THERAPY  HEART_DISEASE  CLINICAL_DEPARTMENT  BLOOD_PRESSURE  DISEASE_SYNDROME  DOSAGE  TREATMENT  TEST  PSYCHOLOGICAL_CONDITION  SYMPTOM  RESPIRATION  LABOUR_DELIVERY  INTERNAL_ORGAN  EXTERNAL_BODY_PART  PROCEDURE | DIABETES  VACCINE  HYPERLIPIDEMIA  HYPERTENSION  DEATH_ENTITY  SYMPTOM  RESPIRATION  LABOUR_DELIVERY  TEMPERATURE  KIDNEY_DISEASE  OBESITY  BMI  PULSE  INJURY |
| **Guidelines for annotations are:**   - *Establish annotation guidelines:* Provide annotators with instructions on how to recognize and label named entities, including the tags to use and rules for determining entity boundaries. - *Use consistent labeling*: To ensure consistency, all annotators should follow the same guidelines and use the same labels. - *Multiple annotators*: Multiple annotators can enhance the reliability and accuracy of the annotations. - *Calculate inter-annotator agreement (IAA)* [1],: This can help assess the consistency of the annotations made by different annotators and identify any areas of disagreement or confusion that may require attention. - *Periodically review and revise guidelines:* As the annotation process progresses, it may be necessary to update the guidelines to ensure they are clear and effective. | | |

**Figure S1**: Active learning for data annotation


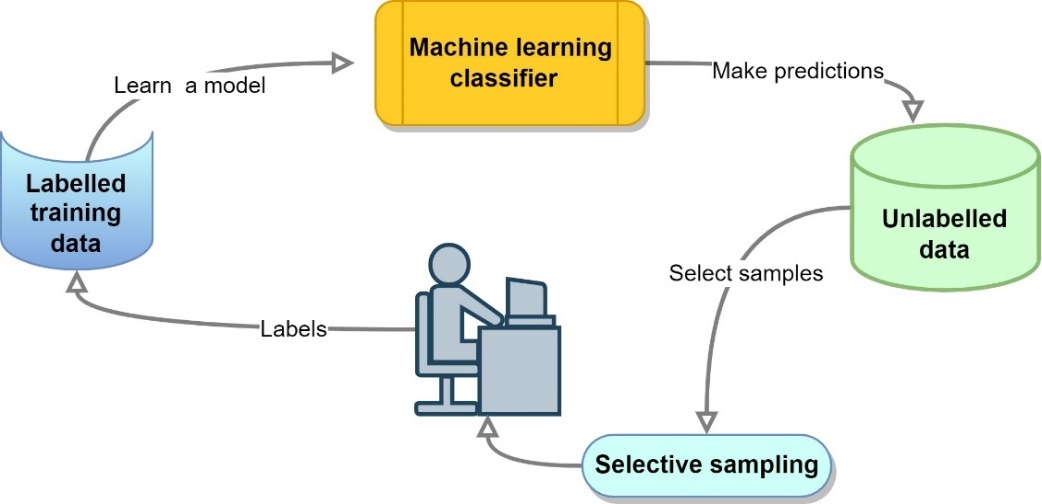


The outline of this active learning approach is: (1) an initial data is taken as a seed (gold labels). (2) BERT was trained using the gold labels (seed) for the token classification. The model performs the predictions on a portion of unlabelled data. The selective sampling [2] technique was used to make a selection from the new predictions, and the selected data samples with new labels were added to the existing initial train set. The classifier was trained on the updated train set, and the accuracy metric was updated. (3) Step 2 was repeated until convergence.

**Figure S2:** Task-specific Transformer model for named entities task


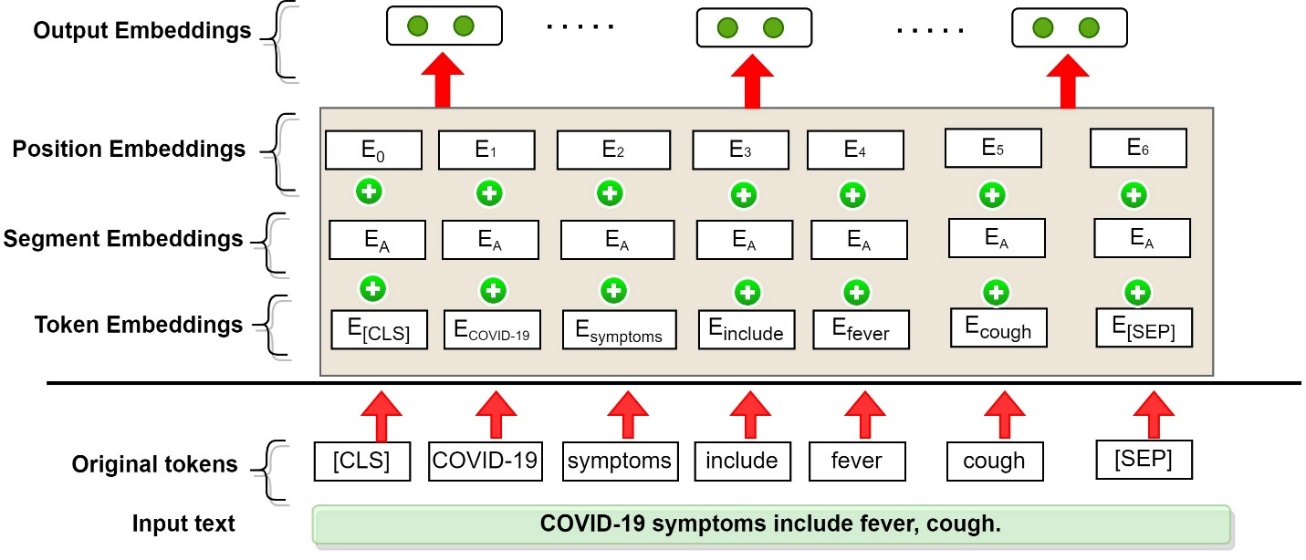


**Table S3:** Notations used in the paper

| **Notation** | **Explanation** |
| --- | --- |
| **Named entity recognition** | |
| $Q$ | Query vector |
| $K$ | Representation vector |
| $V$ | Value vector |
| $d_{k}$ | Input vector dimension. |
| $softmax$ | Activation function |
| $h$ | Number of heads |
| $x_{t}$ | Input text |
| $i_{t}$ , $f_{t}$ and $o_{t}$ | Input, forget and output gate of LSTM unit |
| $C_{t}$, $h_{t}$ | Internal and hidden states |
| *t* | Time |
| $\sigma$ | Activation function sigmoid |
| $tanh()$ | Hyperbolic tangent activation function |
| $W_{i}$, $W_{f}$ $W_{o}$, $W_{C}$, $W_{hi}$ $W_{ho}$,  $W_{hc}$ | Weight matrix of the states |
| $b_{i},b_{f}, b_{o}$, $b_{C}$ | Biases of three gates and a memory cell. |
| $P\left( Y \vert X \right)$ | Conditional probability model in crf |
| *X* | Input variable representing the observation sequence in conditional probability |
| *Y* | Output sequence in crf |
| *f* | Characteristic function |
| $\omega$ | Weight corresponding to the characteristic function |
| **Relation extraction** | |
| $x=[x_{0},x_{1},x_{2}]$ | sequence of token |
| $x_{0}=[CLS]$ | special start |
| $x_{n}=[SEP]$ | end marker |
| $s_{1}=\left( i,j \right)$ and $s_{2}=\left( k,l \right)$ | spans (inline container to mark up a part of a text) |
| $r$ | relation |

**Figure S3:** IOB format by CRF layer**.**


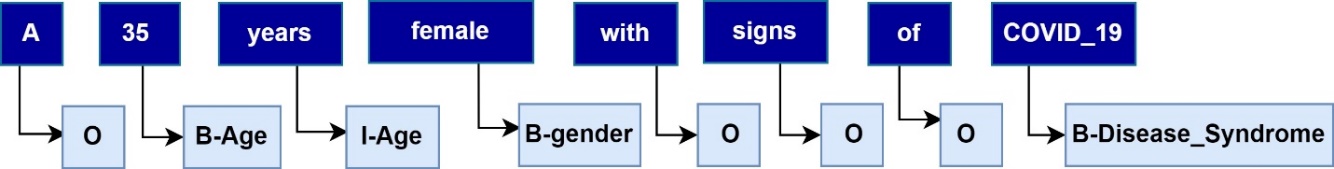


IOB is an entity annotation scheme. The O tag is used for words outside of named entities, while the I-XXX tag is used for words within an XXX-named entity. When two XXX entities are immediately adjacent, the first word of the second entity is tagged B-XXX to indicate that it begins with another entity.

**Table S4:** Case study: *Named entities extracted from the case report (case report text only)*

| **Chunk** | **NER label** | **Confidence (accuracy)** |
| --- | --- | --- |
| 23-year-old | AGE | 0.999 |
| man | GENDER | 1.000 |
| COVID-19 | DISEASE_DISORDER | 0.947 |
| Feb-21 | DATE | 0.870 |
| rRT-PCR | TEST | 0.843 |
| infected | DISEASE_DISORDER | 0.963 |
| He | GENDER | 1.000 |
| infection | DISEASE_DISORDER | 0.981 |
| stress | SYMPTOM | 0.883 |
| In the following days | RELATIVE_DATE | 0.608 |
| he | GENDER | 0.997 |
| fatigue | SYMPTOM | 0.996 |
| shortness of breath | SYMPTOM | 0.592 |
| nocturnal tachycardia | SYMPTOM | 0.520 |
| chest pain | SYMPTOM | 0.832 |
| electrocardiogram | TEST | 0.989 |
| chest x-ray | TEST | 0.827 |
| negative. | TEST | 0.925 |
| his | GENDER | 0.999 |
| he | GENDER | 0.998 |
| attention and memory difficulties | SYMPTOM | 0.457 |
| limb dysesthesia | SYMPTOM | 0.623 |
| Holter ECG | TEST | 0.863 |
| echocardiogram | TEST | 0.980 |
| chest CT | TEST | 0.828 |
| within normal limits | TEST | 0.488 |
| Apr-21 | DATE | 0.917 |
| his | GENDER | 0.998 |
| neurological examination | TEST | 0.513 |
| weakness | SYMPTOM | 0.999 |
| clumsiness of his left arm | SYMPTOM | 0.657 |
| He | GENDER | 1.000 |
| functional movement disorder | DISEASE_DISORDER | 0.238 |
| his | GENDER | 0.999 |
| organic neurological disorder | DISEASE_DISORDER | 0.198 |
| brain MRI | TEST | 0.784 |
| nerve conduction studies | TEST | 0.773 |
| electromyography | TEST | 0.987 |
| evoked potentials | TEST | 0.932 |
| negative | TEST | 0.974 |
| Arm weakness | SYMPTOM | 0.892 |
| slowness | SYMPTOM | 0.380 |
| drift without pronation | SYMPTOM | 0.725 |
| deep tendon | INTERNAL ORGAN | 0.465 |
| weakness | SYMPTOM | 0.992 |
| common functional motor symptoms | SYMPTOM | 0.229 |
| non-motor disturbances | SYMPTOM | 0.690 |
| anxiety | PSYCHOLOGICAL_CONDITION | 0.990 |
| fatigue | SYMPTOM | 1.000 |
| psychiatric disturbances | SYMPTOM | 0.343 |
| mood disorders | PSYCHOLOGICAL_CONDITION | 0.601 |
| pathological personality traits | SYMPTOM | 0.186 |
| SARS-CoV-2 infection | DISEASE_DISORDER | 0.870 |
| Neuropsychological evaluation | TEST | 0.778 |
| normal | TEST | 0.499 |
| cognitive profile | TEST | 0.501 |
| depression | PSYCHOLOGICAL_CONDITION | 0.998 |
| elevated | TEST | 0.299 |
| anxiety levels | TEST | 0.693 |
| FND | DISEASE_DISORDER | 0.687 |
| neurologist | EMPLOYMENT | 0.990 |
| psychoanalytic psychotherapy | TREATMENT | 0.735 |
| targeted physical therapy | TREATMENT | 0.588 |
| a few weeks later | RELATIVE_DATE | 0.576 |
| hospital | CLINICAL_DEPARTMENT | 0.925 |
| he | GENDER | 0.998 |
| post-COVID-19 syndrome | DISEASE_DISORDER | 0.728 |
| After 4 months | RELATIVE_DATE | 0.535 |
| dysesthesia | SYMPTOM | 0.999 |
| motor symptoms | SYMPTOM | 0.659 |
| subjective cognitive complaints | SYMPTOM | 0.297 |
| his | GENDER | 0.995 |
| post-exertional malaise | SYMPTOM | 0.587 |
| chest pain | SYMPTOM | 0.808 |
| muscle tension | SYMPTOM | 0.246 |
| his | GENDER | 0.807 |
| back | EXTERNAL_ORGAN | 0.988 |
| arm | EXTERNAL_ORGAN | 0.826 |

**Figure S4:** Case study, Visual representation of named entities from the snippet of case report.


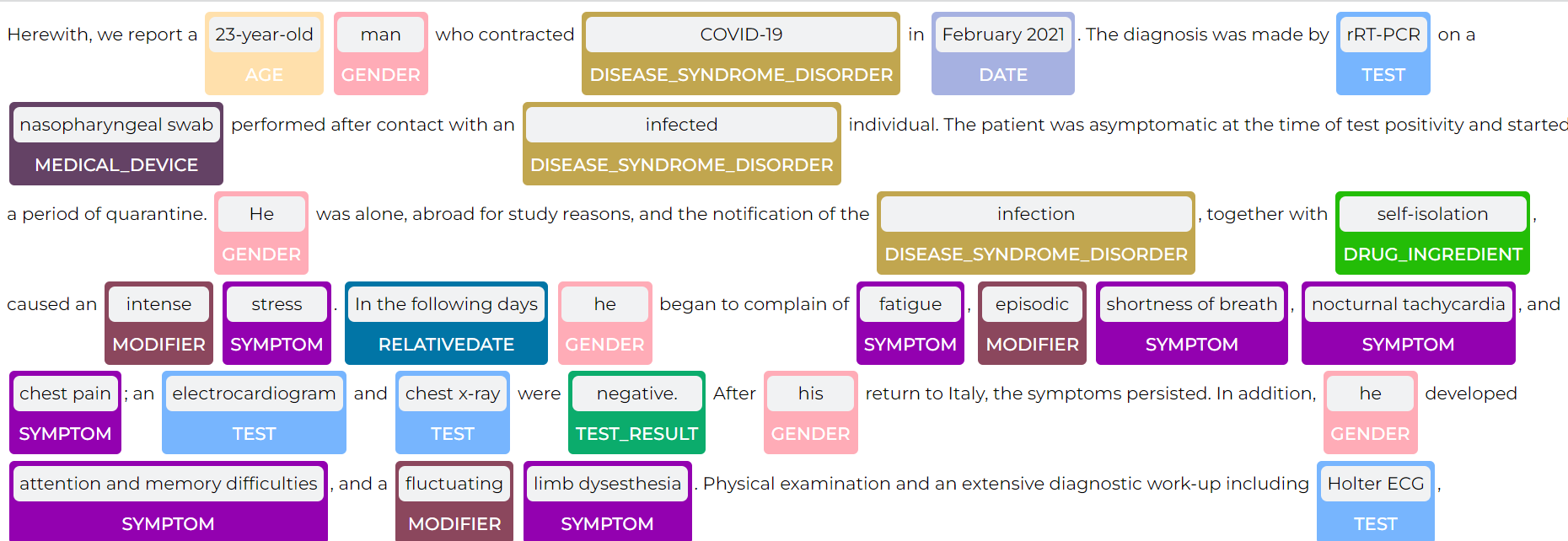


**Table S5: NER on a general case report** [3]**.**

|  | **sentence** | **begin** | **end** | **chunks** | **entity** | **confidence** |
| --- | --- | --- | --- | --- | --- | --- |
| **0** | 0 | 3 | 13 | 37-year-old | Age | 0.9997 |
| **1** | 0 | 15 | 23 | Caucasian | Race | 0.9985 |
| **2** | 0 | 25 | 30 | female | Gender | 0.9996 |
| **6** | 0 | 66 | 77 | palatal mass | Symptom | 0.53505 |
| **7** | 0 | 91 | 108 | breathing problems | Symptom | 0.6171 |
| **8** | 1 | 135 | 140 | trauma | Injury_or_Poisoning | 0.9982 |
| **9** | 1 | 190 | 212 | Total surgical excision | Procedure | 0.59787 |
| **11** | 1 | 228 | 237 | anesthesia | Drug_Name | 0.9966 |
| **12** | 1 | 259 | 287 | histopathological examination | Test | 0.6262 |
| **15** | 2 | 327 | 346 | Immunohistochemistry | Test | 0.682 |
| **16** | 2 | 377 | 381 | tumor | Disease_Syndrome | 0.9963 |
| **18** | 2 | 416 | 427 | fibromatosis | Disease_Syndrome | 0.9504 |
| **21** | 4 | 488 | 503 | Six months after | RelativeDate | 0.77073 |
| **23** | 4 | 537 | 542 | lesion | Symptom | 0.9924 |

**Figure S5:** Dependency parsing.

***
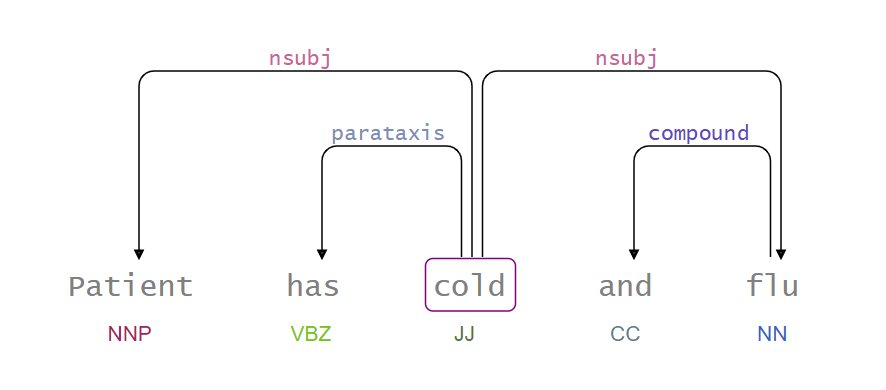
***

These dependencies are explained as: NNP is Proper noun, VBZ is verb, present tense, JJ is adjective, CC is conjunction and NN is noun. More details about the syntactic annotation can be found in the Universal Dependencies guidelines. We use the CoNLL-U annotations from the universal dependencies (U) that provides a standardized set of dependency syntax dataset to train the dependency parser model. The output of the DP is a set of dependencies (usually as a dependency tree) where typed dependency relations connect the words of the input sentence.

**Figure S6:** Relation between disease disorder (entity) and psychological condition (entity).


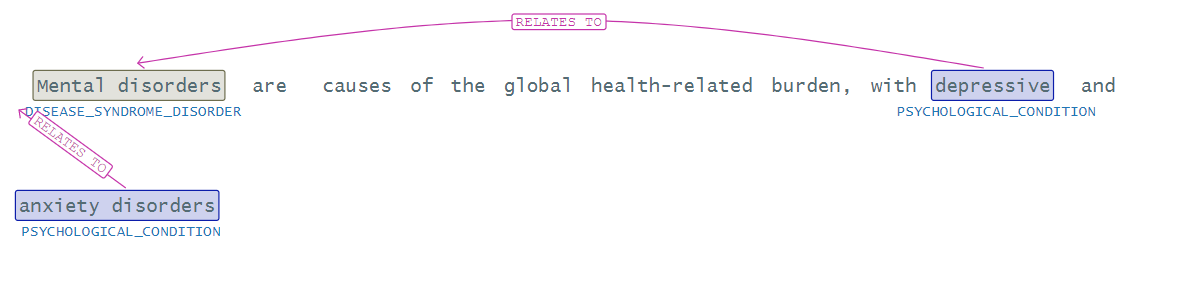


Here, we specify a relation between the disease order and the psychological condition.

**Table S6:** Natural language processing-based summary of COVID-19 cohort.

From our COVID-19 data cohort, we retrieved 4338 case reports, where each case report generally corresponds to one patient report [4], though they may be exceptions. The primary data points we extract from our methods are the patients’ underlying conditions, symptoms, mortality, hospitalizations, and demographic analysis.

| **Factor** | **Value** |
| --- | --- |
| Total number of patients | 4338 |
| Age groups, in years | 6-12 years, 13-18 years, 19-44 years, 45-64 years, 65+ years |
| Male, female, | 45% (n=1952) of patients were male, 31% (n=1334) were female, and 24% (n=1042) could not be identified |
| Source of admission | Operating room  Emergency room  ICU  Other determined as hospital |
| Other disease syndrome diagnosed | Cardiovascular  Cerebrovascular  Respiratory  Long-COVID  Neurological  Multisystem disease  Acute respiratory distress syndrome (ARDS)  Myalgic encephalomyelitis/chronic fatigue syndrome (ME/CFS)  Trauma Other diseases |
| Co-existing disorders with COVID-19 | Cardiovascular disease and hypertension  Chronic Respiratory disease and pulmonary hypertension  Diabetes-Obesity  Diabetes-Hypertension  Diabetes – hyperlipidemia  Hypertension – Obesity  Coronary artery disease (CAD)-Diabetes  Cerebrovascular and Hypertension  Pneumonia and acute respiratory distress syndrome (ARDS)  Other comorbidities |
| Clinical outcomes | Confirmed positive, diagnosed, hospitalization, mortality, recovered, unidentified (shown through figures in Analysis) |

**Table S7:** Benchmark datasets and methods.

|  | | **Corpus** | **Entity types** | **Data size** | |
| --- | --- | --- | --- | --- | --- |
| **NER Task** | | NCBI-Disease [5] | Diseases | 793 PubMed abstracts | |
|  |  | BC5CDR [6] | Diseases | 1500 PubMed articles | |
|  |  | BC5CDR [6] | Chemicals | 1500 PubMed articles | |
|  |  | BC4CHEMD [7] | Chemicals | 10,000 PubMed abstracts | |
|  |  | BC2GM [8] | Gene/Proteins | 20,000 sentences | |
|  |  | JNLPBA [9] | Genes, proteins | 2404 abstracts | |
|  |  | i2b2-Clinical [10] | Problem, Treatment, and Test. | 426 discharge summaries | |
|  |  | I2b2 2012 [11] | Clinical (problems, tests, treatments, clinical departments, occurrences (admission, discharge) and evidence). | 310 discharge summaries | |
| **RE Task** | | ADE [12] | drugs; adverse effects; dosages | 2,972 MEDLINE case report | |
|  |  | BioInfer [13] | Protein-Protein Interaction | 1,098 sentences | |
|  |  | BC5CDR [6] | Chemicals, disease | 1,500 PubMed abstracts | |
|  |  | JNLPBA [9] | Genes, proteins | 2404 abstracts | |
|  |  | CHEMPROT [14] | Protein–chemical | 1,820 PubMed abstracts | |
|  |  | i2b2-Clinical [10] | TrIP, TrWP, TrCP, TrAP, TrAP, TeRP, TeCP, PIP  TrIP: A certain treatment has improved or cured a medical problem  TrWP: A patient’s medical problem has deteriorated or worsened because of or in spite of a treatment being administered  TrCP: A treatment caused a medical problem  TrAP: A treatment administered for a medical problem  TrNAP: The administration of a treatment was avoided because of a medical problem  TeRP: A test has revealed some medical problem  TeCP: A test was performed to investigate a medical problem  PIP: Two problems are related to each other | 426 discharge summaries | |
|  |  | i2b2 2012 | Before, after, simultaneous, begun by, ended by, during, before overlap (Temporal relations defined in 2012 i2b2 Challenge) | 310 discharge summaries | |
|  |  | N2C2 | strength−drug (severity), form−drug (form), dosage−drug (do), frequency−drug (fr), route−drug (route), duration−drug (du), reason−drug (reason), ADR−Drug (adverse) | 288 longitudinal patient records. | |
| **Baseline methods for NER task** | 1. BiLSTM-CRF [15], Bidirectional LSTMs and CRF architecture for NER. 2. BiLSTM-CNN-Char [16], a hybrid (LSTM) and Convolutional Neural Network (CNN) architecture that learns both character-level and word-level features for the NER task. 3. BiLSTM-CRF-MTL [17], a multi-task learning (MTL) framework with BiLSTM-CRF model to collectively use the training data of different types of entities. 4. Att-BiLSTM-CRF [18], an attention (Att) based BiLSTM model with a CRF layer for chemical NER task. 5. Doc-Att-BiLSTM-CRF [19], the Document (Doc)-level Attention (Att)-based BiLSTM-CRF network for disease NER task. 6. BiLSTM-contextualized[20], BiLSTM with contextual string embeddings for sequence labeling. 7. CollaboNet [21], which is a collaboration of deep neural networks, i.e., BiLSTM-CRF and with a single task model trained for each specific entity type. 8. SciBERT, a pretrained language model based on BERT pretrained on a large multi-domain corpus of scientific publications to improve performance on downstream scientific NLP tasks. 9. BLUE [22] , biomedical language understanding evaluation (BLUE) with BERT based pre-training for the biomedical language representation tasks. 10. BioBERT [23], a pre-trained biomedical language representation model for biomedical text mining. We use the following model weights:     - BioBERT-Base v1.0 (+ PubMed 200K) - based on BERT-base-Cased.     - BioBERT-Base v1.1 (+ PubMed 1M) - based on BERT-base-Cased     - BioBERT-Base v1.2 (+ PubMed 1M) - BioBERT-Base v1.1 with LM head. | | | |  |
|  | | **Baseline methods for relation extraction task** | | | |
| **Baseline methods for RE task** | | 1. C4.5 DT [24], a statistical technique to extract causal relations from the texts using c45 decision tree (DT) method. 2. BiLSTM-CRF [25] architecture for RE from biomedical literature. 3. BiLSTM-CNN [26], hybrid architecture of a BiLSTM+CNN model for protein-protien extraction with pretrained word embedding and shortest dependency path embedding. 4. RNNs [25], recurrent neural network (RNN) model to identify protein-protein interaction 5. CMAN [27], a deep cross-modal attention network (CMAN) for joint entity and relation extraction. 6. Adversarial (Adv) training [27], a deep neural network to jointly extracting entities and relations from texts. 7. Multi-att-CNN [28], multi-level attention CNNs (Multi-att-CNN) CNN architecture with two levels of attention to extract causal patterns in heterogeneous contexts. 8. BioBERT [23], We use BioBERT v1.2. 9. BLUE BERT [22] for RE task. | | | |

**Table S8:** Hyperparameter and best result value (values in parenthesis represent the parameter ranges tested)

| **Parameters for NER** | |
| --- | --- |
| **Hyperparameter** | **value** |
| LSTM state size | 200 [200 - 300] |
| Dropout rate | 0.5 [0.2 - 0.7] |
| Epochs | 40 [20- 80] |
| Batch size | 16 [8 - 128] |
| Learning rate (lr) | 1.e-05 [1.e-9 – 1.e-2] |
| lr decay coefficient (po) | 0.005 [0.001, 0.01] |
| Warmup steps | 10,000 |
| Optimizer | ADAM [29], β1=0.9 and β2=0.999 |
| Word dimension | 300 [50 – 450] |
| Hidden size LSTM | 300 |
| Gradient clipping | 5.0 |
| Max seq length | 128 |
| Optimization function | ADAM |
| **General parameters for Transformer-based models** | |
| The general parameters used for fine-tuning transformer-based architectures (BioBERT and others) are maximum sequence length of 128, number of layers as 12, number of attention heads also 12 and embedding size as 768. For different datasets, the fine-tuning takes different hours (2 hours, 3 hours, 4 hours and 10 hours for our dataset). In the NER task, we fixed the length of sentences to 512, whereas, for the RE task, we use a sentence length of 128 in our experiments. | |

**Table S9:** High frequency named entities in case reports

| **Drugs** | **Vaccine** | **Treatments** | **Symptoms** |
| --- | --- | --- | --- |
| Hydroxychloroquine | Pfizer-BioNTech | Isolation | Cough |
| [Paxlovid](https://www.fda.gov/media/155049/download) | Moderna | Wear masks | Nasal congestion |
| [Actemra](https://www.fda.gov/media/150319/download) | AstraZeneca | Vaccination | Running nose/ sneeze |
| Immunomodulators | CoronaVac | Oxygen support | Fatigue |
| Steroid | BBIBP-CorV | Medication | Pains |
| Anti-Epileptic Drugs | Janssen |  | Multi-system disease |
| Amoxicillin |  |  | Shortness of breath |
| Chloroquine |  |  | Respiratory distress |
| Anti-viral |  |  |  |
| Antibodies |  |  |  |

**Figure S7**: Hospitalization, ICU admission, and morality in COVID-19 patients with different age groups.

**References:**

1. Artstein R. Inter-annotator agreement. In: Handbook of linguistic annotation. Springer; 2017. p. 297–313.

2. Dekel O, Gentile C, Sridharan K. Selective sampling and active learning from single and multiple teachers. J Mach Learn Res. 2012;13:2655–97.

3. El-naggar HA, El-Mahallawy YA, Harby MI, Abou Madawi NA. Bilateral collagenous fibroma of the hard palate: a case report and review of the literature. J Med Case Rep. 2023;17:5.

4. Caufield JH, Zhou Y, Garlid AO, Setty SP, Liem DA, Cao Q, et al. A reference set of curated biomedical data and metadata from clinical case reports. Sci data. 2018;5:1–18.

5. Doğan RI, Leaman R, Lu Z. NCBI disease corpus: A resource for disease name recognition and concept normalization. J Biomed Inform. 2014;47:1–10.

6. Li J, Sun Y, Johnson RJ, Sciaky D, Wei C-H, Leaman R, et al. BioCreative V CDR task corpus: a resource for chemical disease relation extraction. Database. 2016;2016.

7. Krallinger M, Rabal O, Leitner F, Vazquez M, Salgado D, Lu Z, et al. The CHEMDNER corpus of chemicals and drugs and its annotation principles. J Cheminform. 2015;7:1–17.

8. Smith L, Tanabe LK, Kuo C-J, Chung I, Hsu C-N, Lin Y-S, et al. Overview of BioCreative II gene mention recognition. Genome Biol. 2008;9:1–19.

9. Collier N, Kim J-D. Introduction to the bio-entity recognition task at JNLPBA. In: Proceedings of the International Joint Workshop on Natural Language Processing in Biomedicine and its Applications (NLPBA/BioNLP). 2004. p. 73–8.

10. Uzuner Ö, South BR, Shen S, DuVall SL. 2010 i2b2/VA challenge on concepts, assertions, and relations in clinical text. J Am Med Informatics Assoc. 2011;18:552–6.

11. Sun W, Rumshisky A, Uzuner O. Evaluating temporal relations in clinical text: 2012 i2b2 challenge. J Am Med Informatics Assoc. 2013;20:806–13.

12. Gurulingappa H, Rajput AM, Roberts A, Fluck J, Hofmann-Apitius M, Toldo L. Development of a benchmark corpus to support the automatic extraction of drug-related adverse effects from medical case reports. J Biomed Inform. 2012;45:885–92.

13. Pyysalo S, Ginter F, Heimonen J, Björne J, Boberg J, Järvinen J, et al. BioInfer: a corpus for information extraction in the biomedical domain. BMC Bioinformatics. 2007;8:1–24.

14. Taboureau O, Nielsen SK, Audouze K, Weinhold N, Edsgärd D, Roque FS, et al. ChemProt: a disease chemical biology database. Nucleic Acids Res. 2010;39 suppl\_1:D367--D372.

15. Lample G, Ballesteros M, Subramanian S, Kawakami K, Dyer C. Neural architectures for named entity recognition. arXiv Prepr arXiv160301360. 2016.

16. Chiu JPC, Nichols E. Named Entity Recognition with Bidirectional LSTM-CNNs. Trans Assoc Comput Linguist. 2016;4:357–70.

17. Wang X, Zhang Y, Ren X, Zhang Y, Zitnik M, Shang J, et al. Cross-type biomedical named entity recognition with deep multi-task learning. Bioinformatics. 2019;35:1745–52.

18. Luo L, Yang Z, Yang P, Zhang Y, Wang L, Lin H, et al. An attention-based BiLSTM-CRF approach to document-level chemical named entity recognition. Bioinformatics. 2018;34:1381–8.

19. Xu K, Yang Z, Kang P, Wang Q, Liu W. Document-level attention-based BiLSTM-CRF incorporating disease dictionary for disease named entity recognition. Comput Biol Med. 2019;108:122–32.

20. Akbik A, Blythe D, Vollgraf R. Contextual string embeddings for sequence labeling. COLING 2018 - 27th Int Conf Comput Linguist Proc. 2018;:1638–49.

21. Yoon W, So CH, Lee J, Kang J. Collabonet: collaboration of deep neural networks for biomedical named entity recognition. BMC Bioinformatics. 2019;20:55–65.

22. Peng Y, Yan S, Lu Z. Transfer learning in biomedical natural language processing: an evaluation of BERT and ELMo on ten benchmarking datasets. arXiv Prepr arXiv190605474. 2019.

23. Lee J, Yoon W, Kim S, Kim D, Kim S, So CH, et al. BioBERT: A pre-trained biomedical language representation model for biomedical text mining. Bioinformatics. 2020;36:1234–40.

24. Girju R. Automatic detection of causal relations for Question Answering. 2003;:76–83.

25. Hsieh Y-L, Chang Y-C, Chang N-W, Hsu W-L. Identifying protein-protein interactions in biomedical literature using recurrent neural networks with long short-term memory. In: Proceedings of the eighth international joint conference on natural language processing (volume 2: short papers). 2017. p. 240–5.

26. Quan C, Luo Z, Wang S. A hybrid deep learning model for protein--protein interactions extraction from biomedical literature. Appl Sci. 2020;10:2690.

27. Zhao S, Hu M, Cai Z, Liu F. Modeling dense cross-modal interactions for joint entity-relation extraction. In: Proceedings of the Twenty-Ninth International Conference on International Joint Conferences on Artificial Intelligence. 2021. p. 4032–8.

28. Wang L, Cao Z, De Melo G, Liu Z. Relation classification via multi-level attention cnns. In: Proceedings of the 54th Annual Meeting of the Association for Computational Linguistics (Volume 1: Long Papers). 2016. p. 1298–307.

29. Kingma DP, Ba JL. Adam: A method for stochastic optimization. In: 3rd International Conference on Learning Representations, ICLR 2015 - Conference Track Proceedings. International Conference on Learning Representations, ICLR; 2015.
